# Structural characterization of macro domain–containing Thoeris antiphage defense systems

**DOI:** 10.1126/sciadv.adn3310

**Published:** 2024-06-26

**Authors:** Yun Shi, Veronika Masic, Tamim Mosaiab, Premraj Rajaratman, Lauren Hartley-Tassell, Mitchell Sorbello, Cassia C. Goulart, Eduardo Vasquez, Biswa P. Mishra, Stephanie Holt, Weixi Gu, Bostjan Kobe, Thomas Ve

**Affiliations:** ^1^ Institute for Glycomics, Griffith University, Southport, QLD 4222, Australia.; ^2^ School of Chemistry and Molecular Biosciences, The University of Queensland, Brisbane, QLD 4072, Australia.; ^3^ Australian Infectious Diseases Research Centre, The University of Queensland, Brisbane, QLD 4072, Australia.; ^4^ Institute for Molecular Bioscience, The University of Queensland, Brisbane, QLD 4072, Australia.

## Abstract

Thoeris defense systems protect bacteria from infection by phages via abortive infection. In these systems, ThsB proteins serve as sensors of infection and generate signaling nucleotides that activate ThsA effectors. Silent information regulator and SMF/DprA-LOG (SIR2-SLOG) containing ThsA effectors are activated by cyclic ADP-ribose (ADPR) isomers 2′cADPR and 3′cADPR, triggering abortive infection via nicotinamide adenine dinucleotide (NAD^+^) depletion. Here, we characterize Thoeris systems with transmembrane and macro domain (TM-macro)–containing ThsA effectors. We demonstrate that ThsA macro domains bind ADPR and imidazole adenine dinucleotide (IAD), but not 2′cADPR or 3′cADPR. Combining crystallography, in silico predictions, and site-directed mutagenesis, we show that ThsA macro domains form nucleotide-induced higher-order oligomers, enabling TM domain clustering. We demonstrate that ThsB can produce both ADPR and IAD, and we identify a ThsA TM-macro–specific ThsB subfamily with an active site resembling deoxy-nucleotide and deoxy-nucleoside processing enzymes. Collectively, our study demonstrates that Thoeris systems with SIR2-SLOG and TM-macro ThsA effectors trigger abortive infection via distinct mechanisms.

## Teaser

Bacteria have two distinct classes of the antiviral system called Thoeris that kill infected cells by different mechanisms.

## INTRODUCTION

Bacteria have developed a highly diverse battery of defense mechanisms to combat infections by phages, which collectively have been coined the “immune system” of bacteria (*1*, *2*). Restriction modification and CRISPR-CAS systems, both of which target and cleave foreign nucleic acids, are most common, but studies over the past 5 years have identified multiple new phage-restricting systems with highly diverse defense mechanisms (*3*–*8*). These include systems that use nucleotides as secondary messengers to mediate defense (*9*–*13*), deplete bacteria of essential nucleotides required for either energy metabolism [e.g., nicotinamide adenine dinucleotide (NAD^+^)] (*9*–*12*, *14*, *15*) or phage replication (e.g., deoxynucleotides) (*16*, *17*), modify nucleotides (*18*), adenosine diphosphate (ADP)–ribosylate viral DNA (*19*), disrupt membrane integrity (*20*, *21*), produce small-molecule inhibitors of phage propagation (*22*–*24*), and rely on reverse transcription of small RNAs (*4*, *25*, *26*).

The Thoeris defense system triggers abortive infection (Abi) upon phage detection (*3*, *11*). It is found in more than 2000 bacterial and archaeal genomes and consists of a single ThsA gene and one or multiple ThsB genes (*3*). The ThsB genes encode Toll/interleukin-1 receptor (TIR) domain–containing proteins responsible for phage detection, while the ThsA gene encodes an Abi-triggering effector protein. Two ThsA effector variants have been reported: a silent information regulator (SIR2) and SMF/DprA-LOG (SLOG) domain–containing effector (ThsA^SIR2-SLOG^) and a transmembrane (TM) and macro domain–containing effector protein (ThsA^TM-macro^).

TIR domains function in immune systems across all domains of life (*27*–*29*). They were first described as scaffolding modules involved in Toll-like receptor and interleukin-1 receptor signaling (*30*, *31*), where they orchestrate signal amplification via formation of higher-order oligomers (*29*, *32*, *33*), but research over the past 6 to 7 years has demonstrated that many TIR domains also have self-association–dependent NAD^+^ cleavage activity (*34*–*37*) and can either deplete cellular NAD^+^ (*9*, *10*, *12*, *15*, *34*) or produce a variety of signaling molecules with immune functions (*11*, *38*–*41*). SIR2 domains function as protein deacetylases, ADP ribosyltransferases, or NAD^+^-depleting enzymes (*14*, *42*, *43*), while macro domains are ADP-ribose (ADPR) recognition domains that can bind ADPR in both its free and protein-linked forms (*44*–*46*). Certain types of macro domains [e.g., poly(ADP-ribose) glycohydrolase–like class, MacroD-type class, terminal ADP-ribose glycohydrolase 1, and viral macro domains] have enzymatic activities and can hydrolyze poly(ADPR) (*47*) or remove ADPR from proteins (*48*–*50*).

The activation mechanism of ThsA^SIR2-SLOG^ Thoeris systems has been characterized in detail. Ofir *et al.* (*11*) demonstrated that phage infections detected by *Bacillus cereus* MSX-D12 and *Bacillus dafuensis* FJAT-25496 ThsB proteins result in the production of a cyclic ADPR (cADPR) isomer, which, in turn, activates the NADase function of the ThsA SIR2 domain to rapidly deplete cellular NAD^+^. The chemical structure of this second-messenger molecule was revealed to contain a 1″-3′ *O*-glycosidic bond linking the two ribose moieties in ADPR, and it was therefore named 3′cADPR (*38*, *39*). A related cADPR isomer with a 1″-2′ *O*-glycosidic bond (2′cADPR), which is produced by some bacterial and plant TIR domains, can also activate ThsA but far less potently than 3′cADPR (*38*, *39*). Structural studies of ThsA^SIR2-SLOG^ proteins from *B. cereus* MSX-D12 and *Streptococcus equi* revealed a conserved 3′cADPR-selective pocket in the SLOG domain and showed that 3′cADPR induces a change in the quaternary structure of ThsA^SIR2-SLOG^, which most likely enables NAD^+^ to access the active site in the catalytic SIR2 domain (*39*, *51*). In contrast to ThsA^SIR2-SLOG^ Thoeris systems, the mechanism of activation has been completely unexplored for Thoeris systems with a ThsA^TM-macro^ effector protein.

In this study, we characterized ThsA^TM-macro^ Thoeris defense systems using a combination of x-ray crystallography, machine learning–based molecular modeling, nuclear magnetic resonance (NMR) spectroscopy, and structure-based mutagenesis. We demonstrate that the macro domain of ThsA^TM-macro^ effectors can bind to ADPR and imidazole adenine dinucleotide (IAD), but not 2′cADPR or 3′cADPR that activate ThsA^SIR2-SLOG^ effectors. We also show that the macro domain of a ThsA^TM-macro^ effector self-aggregates in the presence of ADPR or IAD, and crystal packing analyses of ThsA macro domain structures, combined with mutagenesis and AlphaFold2 modeling of full-length effectors, reveal that the macro domain of ThsA^TM-macro^ effectors form open-ended higher-order oligomers, enabling clustering of its TM domains. We further demonstrate that a TIR domain of a ThsB protein belonging to a ThsA^TM-macro^ Thoeris defense system produces both ADPR and IAD, but not cADPR products, using NAD^+^ as a substrate. Lastly, we identify a ThsB subfamily exclusively associated with ThsA^TM-macro^ effectors, the members of which have an active site configuration resembling deoxynucleotide hydrolases and deoxynucleoside transferases. Collectively, our biochemical and structural data demonstrate that the nucleotide signaling requirements for ThsA^SIR2-SLOG^ and ThsA^TM-macro^ effectors are distinct and suggest that ThsA^TM-macro^ effectors can trigger Abi via membrane perturbation by a mechanism that involves nucleotide-induced oligomerization of its macro domain.

## RESULTS

### *Escherichia coli* ThsA binds to ADPR but not the cADPR isomers 2′cADPR and 3′cADPR

**Fig. 1.**
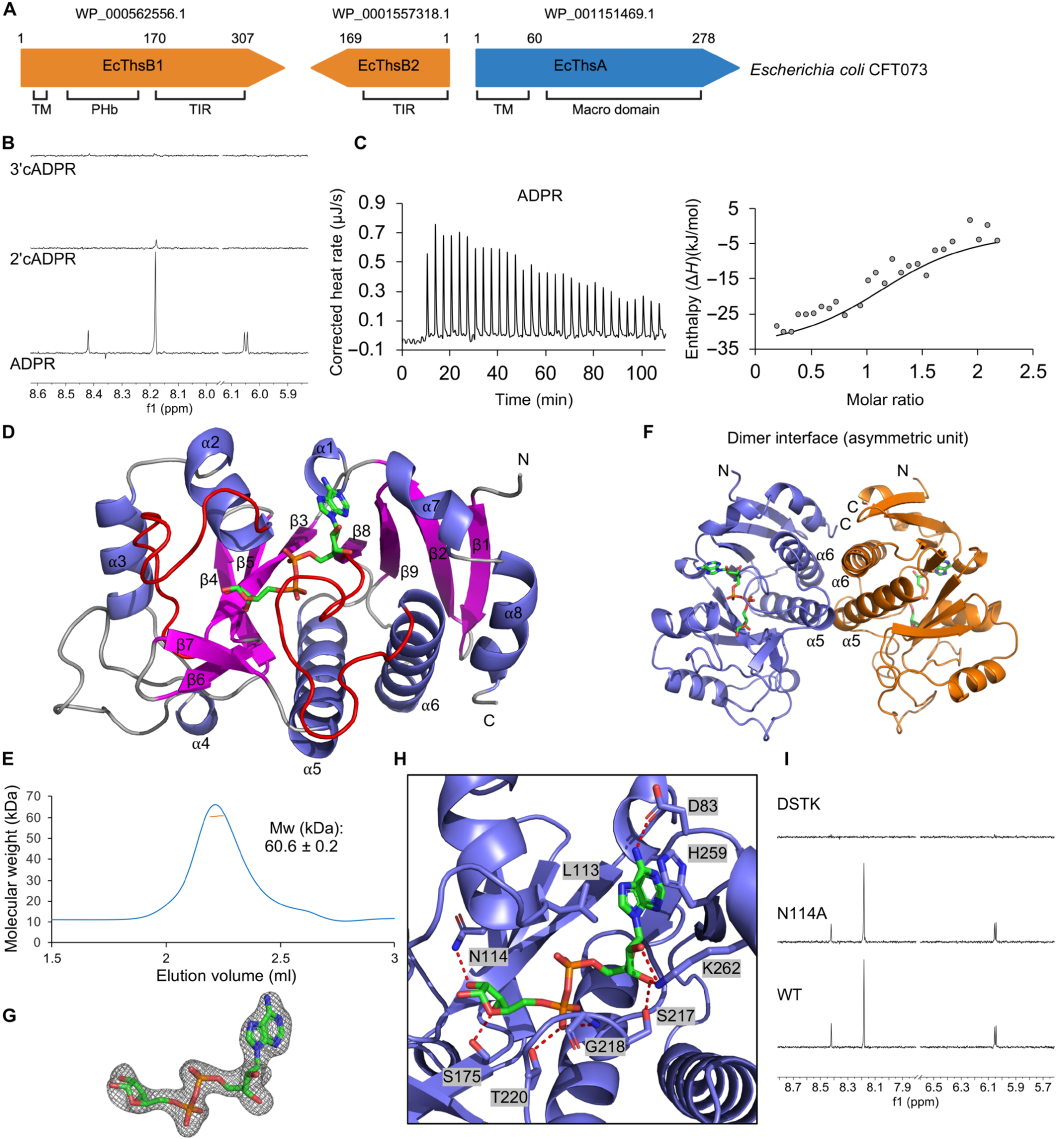
EcThsA^Macro^ binds ADPR. (**A**) Schematic representation of the Thoeris system from *E. coli* CFT073. Protein accession numbers and domains are indicated. PHb, bacterial pleckstrin homology domain. (**B**) Expansions of STD NMR spectra for EcThsA^Macro^ incubated with ADPR, 2′cADPR, and 3′cADPR. Protein concentration was 20 μM, while ligand concentration was 1 mM. (**C**) Raw (left) and integrated (right) ITC data for the titration of 0.3 mM ADPR with 40 μM EcThsA^Macro^. The RQIRKF EcThsA^Macro^ mutant, which does not aggregate in the presence of ADPR, was used for the ITC experiments. (**D**) Ribbon representation of EcThsA^Macro^ crystal structure with secondary structures and the N and C termini labeled. ADPR is shown in stick representation (green). The two ADPR-binding loops are highlighted in red. (**E**) Size exclusion chromatography–coupled multiangle light scattering (SEC-MALS) analysis of EcThsA^Macro^. The blue line represents the refractive index trace, while the orange line represents the average molecular mass distribution across the peak. (**F**) Ribbon representation of the EcThsA^Macro^ dimer observed in the asymmetric unit of the crystal structure. (**G**) Standard omit mFo-DFc map of ADPR, contoured at 3.0 σ. (**H**) Enlarged cutaway of the ADPR binding mode in EcThsA^Macro^. Polar interactions between ADPR and residues of the binding pocket are shown as dashed lines. (**I**) Expansions of STD NMR spectra showing ADPR (1 mM) binding to EcThsA^Macro^ mutants (20 μM). Mw, molecular weight; WT, wild type.

The Thoeris defense system in *E. coli* CFT073 consists of a TM-macro ThsA effector (EcThsA) and two TIR domain–containing ThsB proteins (EcThsB1 and EcThsB2) (Fig. 1A). We expressed and purified the macro domain of EcThsA (EcThsA^Macro^; residues 62 to 278). Binding assays via saturation transfer difference (STD) NMR revealed that EcThsA^Macro^ (20 μM) can bind to ADPR (1 mM), with the aromatic resonances from the adenine moiety of ADPR showing good STD NMR signals (Fig. 1B). No STD NMR signals were detected for the cADPR isomers 2′cADPR and 3′cADPR in the presence of EcThsA^Macro^. Isothermal titration calorimetry (ITC) measurements showed that ADPR binds to EcThsA^Macro^ at a ~1:1 molar ratio, with a dissociation constant (*K*_d_) value of 4.6 ± 0.5 μM (Fig. 1C). No binding was detected for NAD^+^, 2′cADPR, or 3′cADPR using ITC (fig. S1A). To test whether EcThsA has deacetylase and/or glycohydrolase activity, we incubated EcThsA^Macro^ with 2″-*O*-acetyl-ADP-D-ribose (OAADPr) and the alpha anomer of NAD^+^ (α-NAD^+^), respectively. α-NAD^+^ is accepted as a substrate by MacroD macro domains due to its structural resemblance to protein-linked ADPR (*52*, *53*). Real-time NMR assays did not show any deacetylase or glycohydrolase activity for OAADPr and α-NAD^+^, respectively (fig. S1, B and C), indicating that EcThsA^Macro^ is only capable of binding ADPR. Together, these data demonstrate that TM-macro ThsA effectors are nucleotide binding modules with a specificity distinct from SIR2-SLOG ThsA effectors.

### ADPR binds to a highly conserved cleft of EcThsA^Macro^

To define the structural basis for ADPR binding to EcThsA^Macro^, we crystallized and determined structures of this protein in ligand-free state and in complex with ADPR via soaking, at 2.7- and 2.1-Å resolution, respectively (table S1). Ligand density was not observed in crystals soaked with 2′cADPR or 3′cADPR. In agreement with prior sequence analysis, the EcThsA monomer adopts a macro domain fold (Fig. 1D and fig. S2A) with a seven-stranded mixed β sheet sandwiched between eight α helices. The EcThsA^Macro^ structure also features unique insertions: an extended 25-residue loop between the α3 helix and β4 strand and a β-hairpin (β6 and β7 strands) between the β5 and β8 strands (Fig. 1D and fig. S2B). According to DALI structure comparison calculations (*54*), the closest structural relatives are the MacroD-like macro domain from *Oceanobacillus iheyensis* [OiMacroD; Protein Data Bank (PDB): 5L9K; *z* score of 14.9; root mean square deviation (RMSD) of 2.9 Å for 171 Cα atoms; 12% sequence identity] (*55*), macro domain 2 of human ARTD8/PARP14 (PDB: 3VFQ; *z* score of 14.4; RMSD of 2.6 Å for 162 Cα atoms; 10% sequence identity) (*56*), the macro domain of human histone macroH2A1.1 (PDB: 3IIF; *z* score of 14.2; RMSD of 2.5 Å for 159 Cα atoms; 12% sequence identity) (*57*), and the macro domain of AF1521 from *Archaeoglobus fulgidus* (PDB: 2BFQ; *z* score of 14.2; RMSD of 2.6 Å for 165 Cα atoms; 12% sequence identity) (fig. S3A) (*45*). EcThsA^Macro^ exists as a stable dimer in solution, as characterized by size exclusion chromatography–coupled multiangle light scattering (SEC-MALS), and forms a symmetric u-shaped dimer in the asymmetric unit of the crystal (Fig. 1, E and F). The dimer interface involves several hydrophobic residues in the α5 and α6 helices and has a buried surface area of 1840 Å^2^ (Fig. 1F and fig. S2C).

In the crystal structure of EcThsA^Macro^ soaked with ADPR, we observed continuous electron density for a molecule corresponding to ADPR in a highly conserved cleft located above the β3, β8, and β9 strands (Fig. 1, D, G, and H, and fig. S2, D and E). The ADPR binding sites are located on opposite sides of the u-shaped dimer, and binding of ADPR does not lead to substantial structural rearrangements (RMSD of 0.3 Å for 214 Cα atoms) (fig. S2A). The adenine base of ADPR stacks against the side chains of L113 and H259, whereas its N6 nitrogen forms a hydrogen bond with D83 (Fig. 1H and fig. S2E). The C2 and C3 hydroxyls of the proximal adenine-linked ribose form hydrogen bonding interactions with S217 and K262. The pyrophosphate and distal ribose moieties are accommodated between two ADPR-binding loops located between the β3 strand and the α2 helix and the β8 strand and the α6 helix. The pyrophosphate moiety is stabilized via interactions with the G218 backbone amide and T220 side chain, while the distal ribose in the EcThsA^Macro^:ADPR complex is stabilized via hydrogen bonds with the N114 and S175 side chains. The distal ribose conformation is different from ADPR complexes of the MacroD-like family of macro domains (e.g., OiMacroD and AF1521), which hydrolyze protein-ADPr and acyl-ADPr ester bonds, and the MacroH2A-like family of macro domains (e.g., ARTD8/PARP14 and macroH2A1.1), which bind ADPR metabolites and ADPR ribosylated proteins but have no catalytic activity (fig. S3B). Furthermore, unlike the open binding site in the OiMacroD, AF1521, ARTD8/PARP14, and macroH2A1.1 structures, the distal ribose in EcThsA^Macro^ is buried by the unique β_6_-β_7_ hairpin inserted between the β5 strand and the α5 helix (fig. S3C).

Mutational analysis confirmed the importance of EcThsA^Macro^ binding cleft residues for ADPR binding (Fig. 1I). A quadruple EcThsA^Macro^ mutant with the binding cleft residues D83, S217, T220, and K262 (DSTK) mutated to alanines is not capable of binding ADPR. A double alanine mutant of residues equivalent to D100 and N114 in EcThsA^Macro^ has previously been shown to render a macro domain–containing Thoeris system from *Bacillus amyloliquefaciens* Y2 (BaThs) inactive (*3*). As described above, N114 stabilizes the distal ribose moiety of ADPR in EcThsA^Macro^. D100 is not involved in ADPR interactions but stabilizes the unique loop inserted between the α_3_ helix and β_4_ strand by forming hydrogen bonds with the side chains of R150 and N96 and the backbone amide nitrogen of K154 (fig. S2B). These residues are also conserved in the BaThs system, and AlphaFold2 predicts similar interactions for BaThsA (UniProt/AlphaFold Database: I2C645). We made single alanine mutations of EcThsA^Macro^ N114 and D100. The N114A mutant could still bind ADPR (Fig. 1I), but the STD NMR signal intensities were different compared to that with wild-type EcThsA^Macro^, which is consistent with its role in forming a hydrogen bond with the C2 hydroxyl of the distal ribose in ADPR. The D100A mutant was not expressed in a soluble form in *E. coli*, suggesting that maintaining the α_3_-β_4_ loop configuration is critical for the stability of EcThsA^Macro^.

### ThsA macro domains bind to IAD with stronger affinity than to ADPR

**Fig. 2.**
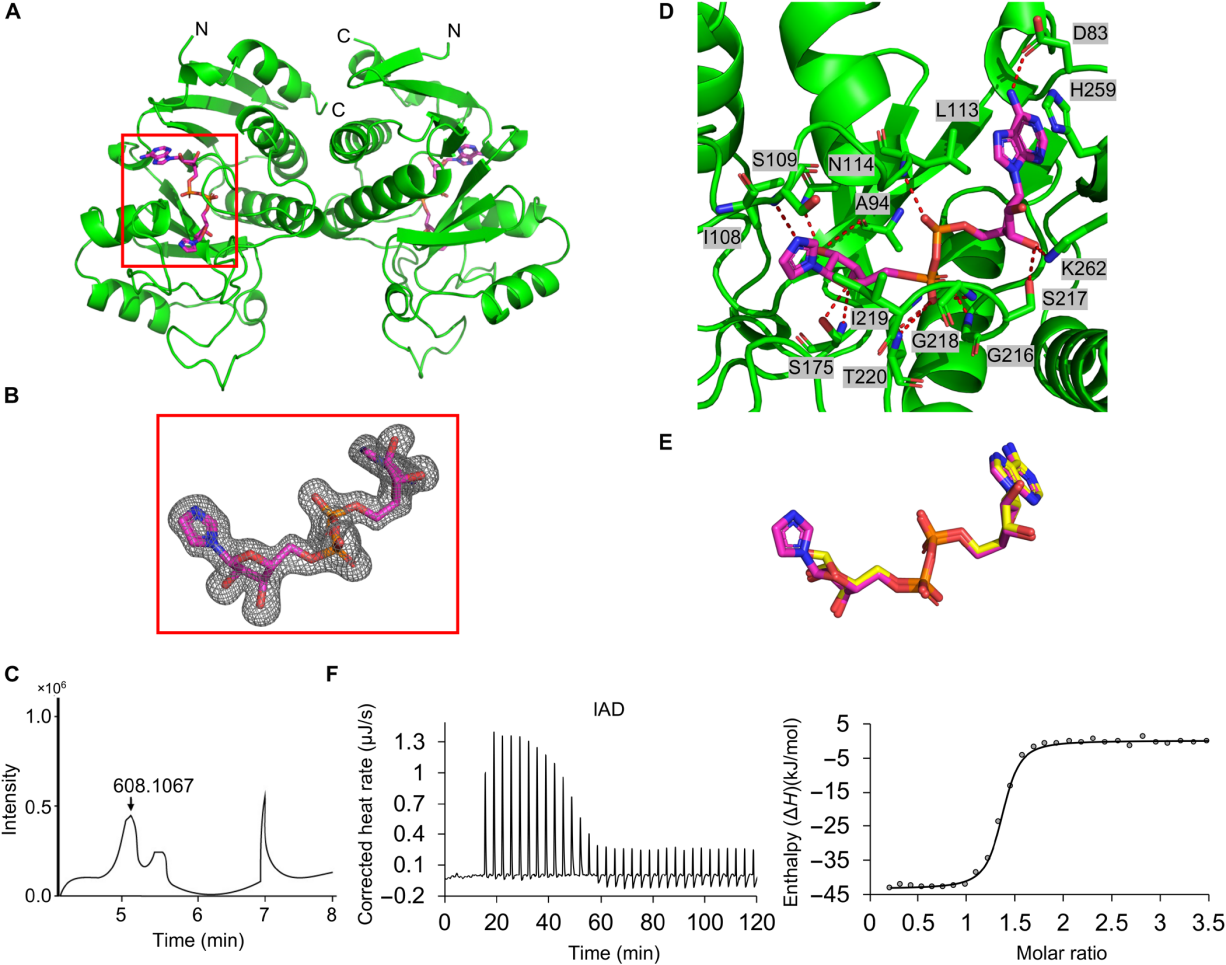
ThsA macro domains bind IAD. (**A**) Ribbon representation (green) of the PcThsA^Macro^ crystal structure with secondary structures and N and C termini labeled. IAD is shown in stick representation (magenta). (**B**) Standard omit mFo-DFc map of IAD, contoured at 3.0 σ. (**C**) LC-MS analysis of small molecules bound to PcThsA^Macro^. (**D**) Enlarged cutaway of the IAD binding mode in PcThsA^Macro^. Polar interactions between IAD and residues of the binding pocket are shown as dashed lines. (**E**) Comparison of binding modes of ADPR (yellow) and IAD (magenta). (**F**) Raw (left) and integrated (right) ITC data for the titration of 0.5 mM IAD with 44 μM EcThsA^Macro^. The RQIRKF EcThsA^Macro^ mutant was used for the ITC experiments.

We also expressed, purified, and determined the crystal structure of the macro domain of *Pseudomonas corrugata* ThsA (PcThsA^Macro^; residues 63 to 278; 66.7% sequence identity with EcThsA^Macro^) at 1.6-Å resolution (table S1). PcThsA^Macro^ also exists as a u-shaped dimer, and the structure is very similar to EcThsA^Macro^, with an RMSD of 0.9 Å for 215 Cα atoms (Fig. 2A). Unexpectedly, in the final map, we observed unambiguous electron density for an adenine dinucleotide with the distal ribose linked to a five-membered ring in the ligand binding pocket (Fig. 2, A and B). Liquid chromatography–mass spectrometry (LC-MS) revealed that the PcThsA^Macro^ bound nucleotide has a mass corresponding to IAD (Fig. 2C). The imidazole base of IAD stacks against the side chains of I108 and I219 while forming a hydrogen bond with the backbone amide nitrogen of S109 (Fig. 2, D and E). ITC measurements show that high-performance liquid chromatography (HPLC)–purified IAD (fig. S4, A to C) binds more strongly to EcThsA^Macro^ than ADPR, with a *K*_d_ value of 167 ± 91 nM (Fig. 2F), consistent with the additional interactions observed in the PcThsA^Macro^:IAD complex. We could not detect IAD in nucleotide extractions from PcThsA^Macro^ purified using a lysis buffer without imidazole present (fig. S4D), suggesting that IAD is produced from imidazole by an endogenous enzyme while preparing the *E. coli* cells for PcThsA^Macro^ purification. In summary, these data reveal the structural basis for nucleotide recognition by TM-macro domain–containing ThsA effectors and demonstrate a preference for IAD over ADPR.

### EcThsA^Macro^ self-aggregates upon ADPR and IAD binding

**Fig. 3.**
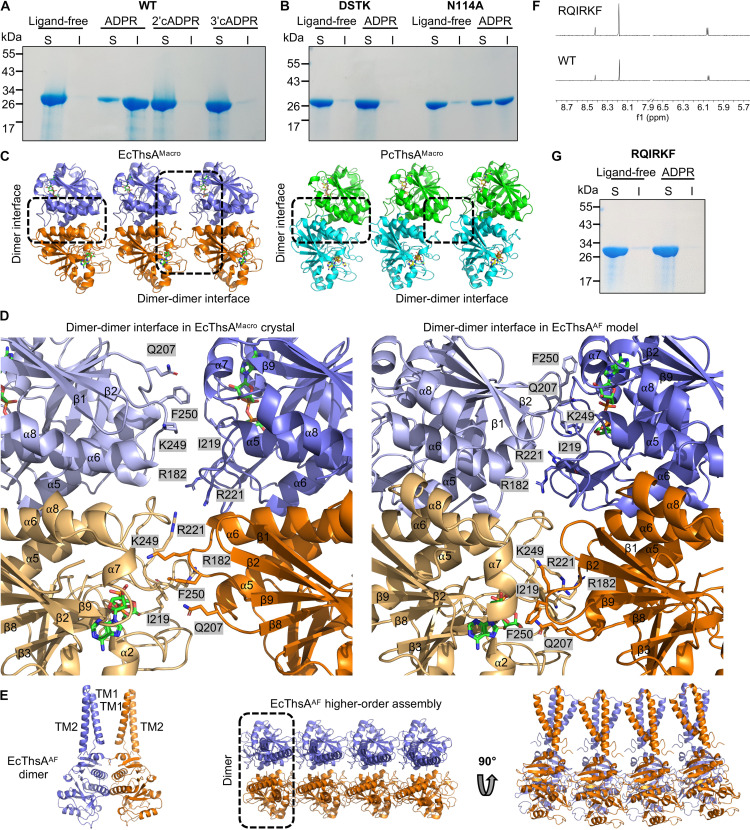
EcThsA^Macro^ self-aggregates upon ligand binding. (**A**) SDS-PAGE analysis of the soluble (S) and insoluble (I) fractions after incubation of EcThsA^Macro^ (47 μM) with ADPR (1 mM), 2′cADPR (1 mM), and 3′cADPR (1 mM) at 4°C for 1 hour. (**B**) SDS-PAGE analysis of the soluble (S) and insoluble (I) fractions after incubation of EcThsA^Macro^ ADPR binding-site mutants (47 uM) with ADPR (1 mM) at 4°C for 1 hour. (**C**) EcThsA^Macro^ and PcThsA^Macro^ crystal packing analyses defines a higher-order oligomerization interface. (**D**) Enlarged cutaways of the dimer-dimer interfaces in the EcThsA^Macro^ crystal structure (left) and EcThsA AlphaFold2 (EcThsA^AF^) model (right). (**E**) Ribbon representations of the EcThsA^AF^ dimer and higher-order oligomer. (**F**) Expansions of STD NMR spectra showing ADPR (1 mM) binding to an EcThsA^Macro^ dimer-dimer interface mutant (20 μM). (**G**) SDS-PAGE analysis of the soluble (S) and insoluble (I) fractions after incubation of wild-type EcThsA^Macro^ and a dimer-dimer interface mutant (47 μM) with ADPR (1 mM) at 4°C for 1 hour. The data in (A), (B), and (G) are representative of three independent experiments (using the same protein batches).

At high protein concentrations (>50 μM), we observed that EcThsA^Macro^ solutions became turbid in the presence of either 1 mM ADPR or 1 mM IAD, suggesting self-aggregation triggered by ligand binding. SDS–polyacrylamide gel electrophoresis (SDS-PAGE) analysis revealed that more than 50% of EcThsA was present in the insoluble fraction after incubation with ADPR or IAD (Fig. 3A and fig. S5A). Negative-stain electron microscopy (EM) visualization revealed that these assemblies are heterogeneous; ordered aggregates such as filaments were not detected (fig. S5B). The DSTK mutant, which abolishes ADPR binding, did not aggregate in the presence of ADPR, while the N114A mutant, which still binds ADPR, aggregated (Fig. 3B). Furthermore, EcThsA^Macro^ solutions incubated with 2′cADPR or 3′cADPR remained clear, and the protein was not detected in the insoluble fraction by SDS-PAGE analysis (Fig. 3A), demonstrating that only ADPR and IAD can trigger self-aggregation of EcThsA^Macro^.

Considering ADPR and IAD binding trigger self-aggregation of EcThsA^Macro^ in solution, we next examined the packing within the EcThsA^Macro^ and PcThsA^Macro^ crystal structures for potential higher-order oligomeric interfaces. In both crystals, the symmetric u-shaped dimers stack against each other, forming open-ended higher-order oligomers (Fig. 3C). In EcThsA^Macro^, the dimer-dimer interfaces are predominantly composed of residues from the β6-β7 hairpin and the β3-α2, β8-α6, α5-β8, and α6-β9 loops, with a total buried surface area of 1060 Å^2^ (Fig. 3, C and D). The ADPR/IAD binding sites are located at the dimer-dimer interfaces, but neither ADPR nor IAD is directly involved in subunit interactions. In PcThsA^Macro^, the dimer-dimer interfaces are smaller, with a total buried surface area of 840 Å^2^, and they predominantly involve residues from the α6-β9 loops (Fig. 3C and fig. S5C). In both structures, the N termini of the macro domains are localized on the same side of these linear assemblies, which is compatible with oligomerization along the two-dimensional surface of the bacterial cell membrane. Using AlphaFold2 multimer, we constructed dimeric and oligomeric (octamer) models of full-length EcThsA (EcThsA^AF^) and PcThsA (PcThsA^AF^) (Fig. 3E and figs. S5D and S6). Notably, the AlphaFold2-predicted structures closely match the macro domain dimer and oligomer observed in the EcThsA^Macro^ crystal structure (Fig. 3, C to E, and fig. S5, C to E), but the dimer-dimer interfaces are considerably more extensive in the AlphaFold2 models, with a buried surface area of 3220 to 3400 Å^2^ (Fig. 3D and fig. S5E). The TM domains protrude from the top of the u-shaped macro domain dimers forming loosely connected TM domain assemblies (Fig. 3E and fig. S5D). While TM helix 2 is predominantly hydrophobic, TM helix 1 is predicted to have one hydrophobic face and one hydrophilic face (fig. S5F), and we speculate that the TM domains of ThsA can promote formation of membrane lesions or pores upon oligomerization.

To validate the dimer-dimer interface observed in the crystal and AlphaFold2 models, we designed an EcThsA^Macro^ mutant (RQIRKF) with six interface residues mutated to glutamate or alanine (R182E, Q207E, I219A, R221E, K249E, and F250A). As expected, the mutant could still bind ADPR and IAD (Figs. 1C, 2F, and 3F) but without self-aggregation (Fig. 3G), validating our structural model. The ADPR/IAD binding pocket is located at the dimer-dimer interface in the EcThSA^Macro^ crystal structure and the ThsA^TM-macro^ AlphaFold2 models, but neither ADPR nor IAD interacts directly with other macro domain subunits in these assemblies. We speculate that ADPR and IAD can lower the critical concentration for higher-order oligomerization by modulating or stabilizing the β3-α2 loop, the β8-α6 loop, and/or the β6-β7 hairpin, which are involved in dimer-dimer interactions in the crystal structures and Alphafold2 models. In summary, our data support a mechanism where ThsA^TM-macro^ exists as an inactive dimer and subsequently oligomerizes within the membrane upon nucleotide binding.

### EcThsB1 catalyzes NAD^+^ hydrolysis and base-exchange reactions

**Fig. 4.**
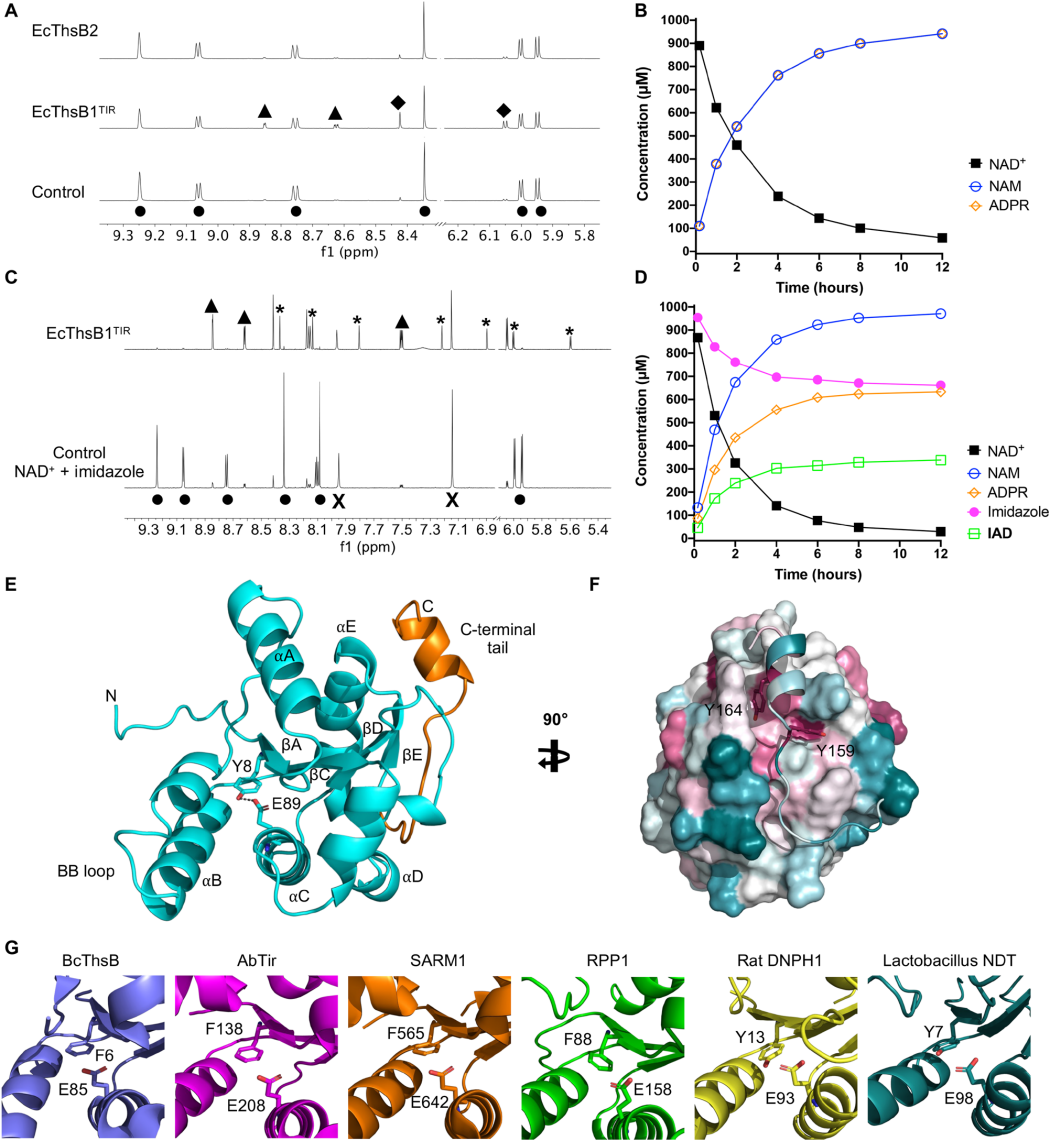
EcThsB1 NADase activities and EcThsB2 crystal structure. (**A**) Left: Expansions of ^1^H NMR spectra showing NADase activity for the EcThsB1^TIR^ but not EcThsB2. The initial NAD^+^ concentration was 1 mM, and the protein concentration was 10 μM. Spectra correspond to 64-hour incubation time, except for the control. Selected peaks are labeled, showing the production of nicotinamide (NAM) (▲) and ADPR (◆) from NAD^+^ (●). (**B**) Reaction progress curves of 0.25 μM EcThsB1^TIR^ + 1 mM NAD^+^. (**C**) Expansions of ^1^H NMR spectra showing imidazole base-exchange reaction by 0.25 μM EcThsB1^TIR^, with a no-protein control sample of 1 mM NAD^+^ + 1 mM imidazole (bottom) and a sample of 0.25 μM EcThsB1^TIR^ + 1 mM NAD^+^ + 1 mM imidazole incubated for 12 hours (top). Selected peaks are labeled for NAD^+^ (●), ADPR (◆), NAM (▲), imidazole (X), and IAD (*). (**D**) Reaction progress curves of 0.25 μM EcThsB1^TIR^ + 1 mM imidazole + 1 mM NAD^+^. (**E**) Ribbon representation of EcThsB2 crystal structure (cyan), with secondary structures and N and C termini labeled. The C-terminal tail is highlighted in orange. The active site residues Y8 and E89 are shown in stick representation. (**F**) Surface representation of EcThsB2, highlighting the interaction of the C-terminal tail with the TIR domain surface. The surface was colored by sequence conservation using ConSurf (*94*). Cyan corresponds to variable regions, while purple corresponds to conserved regions. (**G**) Comparison of the active site in TIR domains from *B. cereus* ThsB (BcThsB; PDB: 6LHY), AbTir (PDB: 7UXU), SARM1 (PDB: 6O0R) and *Arabidopsis thaliana* resistance protein RPP1 (PDB: 7DFV) (*36*, *39*, *51*, *95*), with rat DNPH1 (PDB: 4FYH) (*96*) and *Lactobacillus* NDT (PDB: 1F8X) (*59*).

SIR2-SLOG ThsA effectors are activated by 3′cADPR, produced by ThsB proteins. To identify the nucleotide products generated by ThsB proteins of Thoeris systems with a TM-macro ThsA effector, we expressed and purified the TIR domains of EcThsB1 (EcThsB1^TIR^) and EcThsB2. Real-time NMR-based NADase assays revealed that EcThsB1^TIR^ (but not EcThsB2^TIR^, see below) could use NAD^+^ as a substrate to produce nicotinamide and ADP ribose (ADPR), but not the cADPR isomers 2′cADPR or 3′cADPR (Fig. 4, A and B), consistent with previous observations (*35*). Next, we tested whether EcThsB1^TIR^ can catalyze base-exchange reactions with heterocyclic amines, which has recently been reported for the TIR domains of human sterile alpha and TIR motif–containing 1 (SARM1) and *Acinetobacter baumannii* TIR (AbTir) (*39*, *58*). We observed that EcThsB1^TIR^ can indeed catalyze NAD^+^ base-exchange reactions with pyridine and multiple pyridine-fused heterocycles (fig. S7, A to D). Because PcThsA^Macro^ and EcThsA^Macro^ can bind to IAD, we also tested several five-membered heterocyclic compounds (imidazole, thiazole, and pyrazole), histidine, histamine, and adenine, which consists of imidazole and pyrimidine rings fused together. Of these compounds, only imidazole, histamine, and thiazole were observed to undergo base-exchange reactions with NAD^+^ in the presence of EcThsB1^TIR^ (Fig. 4, C and D, and fig. S7, A to D).

In the presence of a biochemically stable NAD^+^ mimetic, 8-amino-isoquinoline adenine dinucleotide (**3AD**), we were able to visualize filamentous structures of EcThsB1^TIR^ by negative-stain EM (fig. S8A), suggesting that the active site is formed upon TIR domain self-association as observed for SARM1 and AbTir^TIR^ (*39*, *58*). Furthermore, AlphaFold2 multimer predicts that EcThsB1^TIR^ forms two-stranded parallel TIR domain assemblies as observed in the cryo-EM structure of the AbTir^TIR^ filament (PDB: 7UXU) (fig. S8B) (*39*). Our results further support the hypothesis that adenine dinucleotides with an imidazole-containing base are signaling molecules of Thoeris defense systems with a TM-macro ThsA effector.

### EcThsB2 has an active site tyrosine only found in Thoeris defense systems with a TM-macro ThsA effector

We did not detect NADase activity for EcThsB2 (Fig. 4A), but its crystal structure revealed a unique active site configuration with a tyrosine residue within hydrogen bonding distance to the catalytic glutamate residue (Fig. 4, E and F, and table S1). TIR domains with NADase activity from bacteria, plants, and animals usually have a phenylalanine or alanine in the position equivalent to this tyrosine (fig. S9). Similar active sites, with a tyrosine residue forming a hydrogen bond with the catalytic glutamate, are observed in nucleoside 2′-deoxyribosyltransferases (NDTs) and the 2′-deoxynucleoside 5′-phosphate *N*-hydrolase 1 (DNPH1) (Fig. 4G and fig. S10, A and B). These enzymes catalyze the cleavage of the glycosidic bond in various nucleosides and nucleotides and have very high degrees of specificity toward 2′-deoxy-nucleosides and 2′-deoxy-nucleotides (*59*, *60*). In the presence of both a tyrosine and 2′-hydroxyl–containing substrate, it is likely that the glutamate in these enzymes becomes too constrained by hydrogen bonds to efficiently catalyze the cleavage of the glycosidic linkage (*61*).

**Fig. 5.**
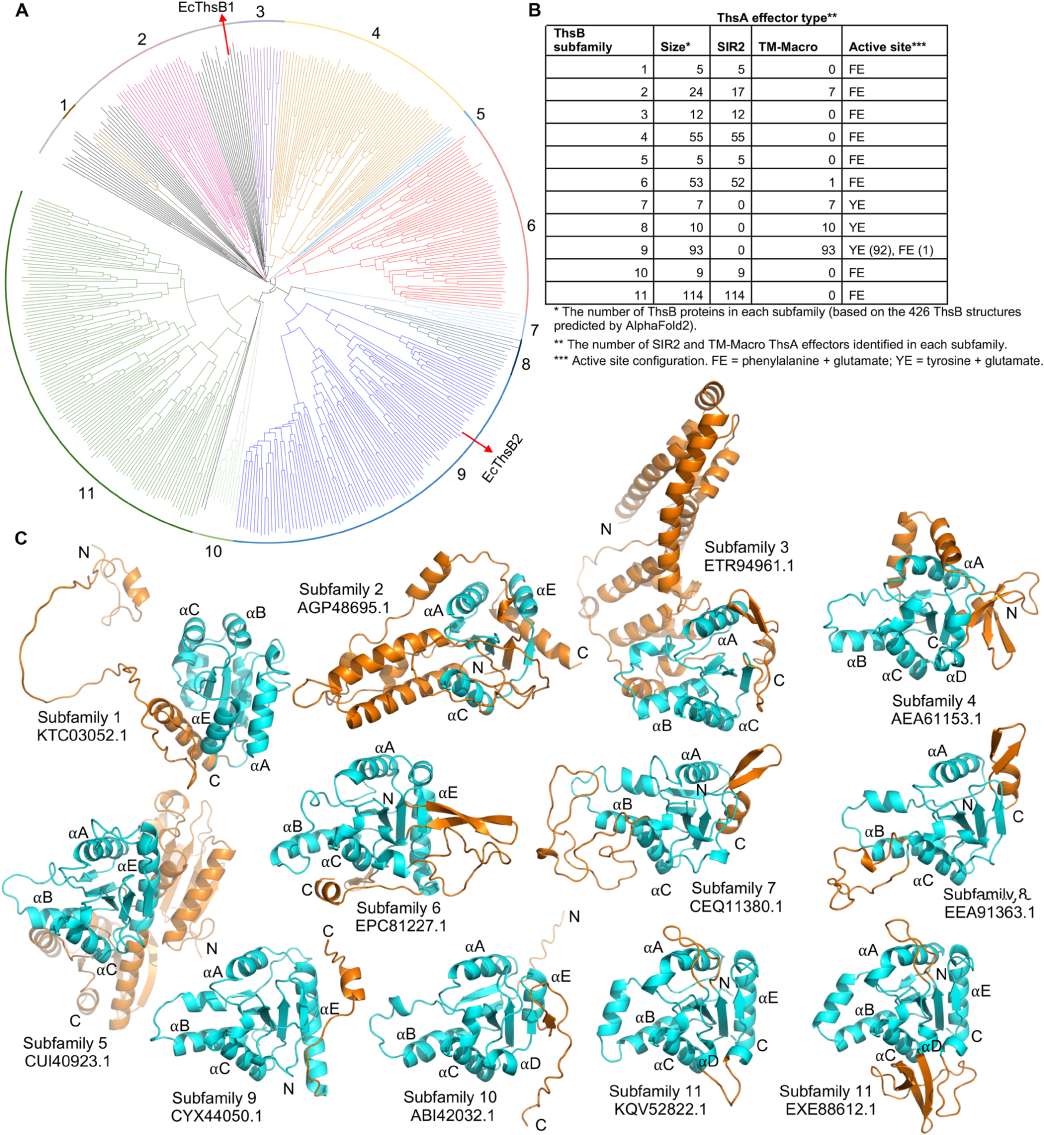
AlphaFold2-predicted structures of ThsB proteins. (**A**) Structure-based phylogenetic tree of 426 AlphaFold2-predicted ThsB structures generated using Foldtree (*89*). On the basis of the tree and structure alignment analyses in PyMOL, 11 ThsB subfamilies (with ≥5 structures) were identified. Structures classified into subfamilies are shown in color. The remaining 39 ThsB structures, which were not included in any subfamilies, are highlighted in black. *E. coli* ThsB proteins characterized in this study (EcThsB1 and EcThsB2) are denoted with red arrows. (**B**) Summary table of the 11 ThsB subfamilies showing their size, ThsA effector type, and active site configuration. (**C**) Representative structures of the ThsB subfamilies are shown in ribbon representation. The TIR domains are shown in cyan with loop insertions and N- and C-terminal extensions, and additional domains are highlighted in orange. Two structures have been included for ThsB subfamily 11 to highlight the structural diversity observed in the αD helical region.

AlphaFold2 modeling and subsequent analysis of 426 ThsB proteins (≤90% sequence identitiy) from Doron *et al.* (*3*) revealed that ThsB proteins with an active site tyrosine form three subfamilies that are exclusively found in Thoeris defense systems with a TM-macro ThsA effector (Fig. 5, fig. S11, and table S2). The subfamily containing EcThsB2 is the second largest ThsB subfamily in this dataset (subfamily 9 in Fig. 5; ThsB_subfamily 9_) and comprises 73.8% of the predicted ThsB structures from Thoeris defense systems with a TM-macro ThsA.

EcThsB2 and ThsB_subfamily 9_ members also feature a ~20-residue C-terminal tail that folds back onto the TIR domain (Figs. 4, E and F, and 5C), which is likely to interfere with TIR domain self-association to create the active site configuration observed in SARM1 and AbTir (fig. S12). To test whether this tail affects ThsB activation, we generated two variants of EcThsB2: a modified tail with Y159 and Y164 replaced by alanines (EcThsB2^Y159A, Y164A^) and a tail deletion (EcThsB2^Δ151–169^). However, neither of the variants could be expressed in a soluble form in *E. coli*, suggesting that this tail may also be required for proper folding of EcThsB2. Together, these structural analyses identify a class of TIR domains with a unique active site configuration associated with TM-macro Thoeris systems.

## DISCUSSION

**Fig. 6.**
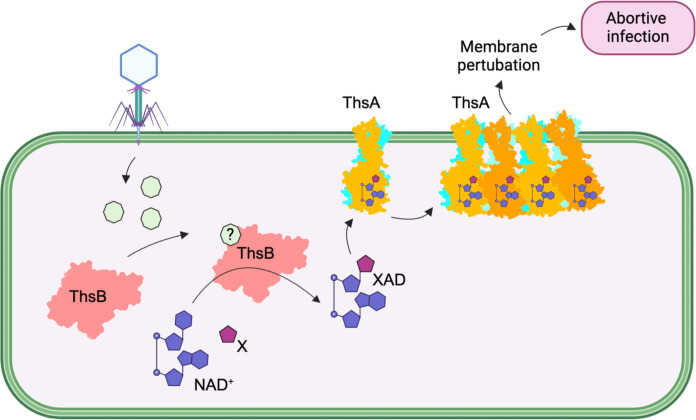
Model for the mechanism of action of Thoeris defense systems with a TM and macro domain containing ThsA effector. X represents a (heterocyclic) base cosubstrate of ThsB-catalyzed base-exchange reactions; XAD represents adenine dinucleotides formed via ThsB-catalyzed base-exchange reactions. The figure was created with BioRender (www.biorender.com).

TIR domains produce highly diverse immune signals in response to pathogen challenge using NAD^+^ as a substrate (*27*, *28*, *38*–*41*, *62*, *63*). Here, we show that *E. coli* Thoeris defense systems with TM-macro ThsA effector utilize a distinct set of nucleotides for defense activation, compared to SIR2-SLOG ThsA effectors that are activated by 3′cADPR. Our structural characterization of TM-macro ThsA proteins additionally suggests a mechanism for how these effectors can trigger an immune response upon nucleotide binding (Fig. 6).

Macro domains are evolutionarily conserved structural modules that play key roles in the recognition, interpretation, and turnover of ADPR signaling. They are found in all domains of life and are also abundant in viruses, including phages (*64*). Our structural and biochemical data suggest that the nucleotide binding pocket in ThsA macro domains is selective for ADPR and adenine dinucleotides and cannot bind to or process ADPR derivatives such as poly(ADPR), mono-ADP ribosylated proteins, or OAADPr, which are common ligands of many macro domains (*64*). Our data also demonstrate that ThsA macro domains exist as stable dimers in solution and further oligomerize upon ADPR binding. To our knowledge, dimerization and higher-order oligomerization have so far not been reported for any other macro domain and thus appear to be unique features of Thoeris defense system–associated macro domains. It will be of interest to determine whether other macro domains involved in antiphage defense also feature nucleotide-induced oligomerization. The open-ended nature of the predicted higher-order ThsA^TM-macro^ oligomers may support formation of large-scale membrane-disrupting TM domain clusters, as recently reported for cyclic oligonucleotide-based antiphage signaling system (CBASS) TM effectors (*20*). These CBASS effectors also oligomerize upon nucleotide binding and have a similar two-helix TM domain architecture to ThsA proteins. Future structural studies of full-length ThsA^TM-macro^ effectors will define exactly how ligand binding induces higher-order oligomerization and whether TM domain clustering results in membrane disruption.

We show that IAD is a considerably stronger (~30-fold) ThsA^Macro^ ligand than ADPR. Although both PcThsA^Macro^ and EcThsA^Macro^ were purified using the same method with imidazole-containing buffers, IAD only copurified with PcThsA^Macro^. Considering that IAD induce aggregation of EcThsA^Macro^, we speculate that EcThsA:IAD complexes formed inside the bacterial cell or, after cell lysis, precipitated during lysate clarification.

We also show that a ThsB TIR domain from a ThsA^TM-macro^ Thoeris system can produce IAD using imidazole and NAD^+^ as substrates via a base-exchange reaction. Furthermore, AlphaFold2 modeling reveal that many ThsB proteins (Fig. 5; subfamilies 7 to 9) found in Thoeris systems with a ThsA^TM-macro^ effector have an active site configuration closely related to the NDT family of enzymes, which catalyze base-exchange reactions between the purine or pyrimidine bases of 2′-deoxyribonucleosides and free pyrimidine or purine bases (*65*). Together, these results suggest that Thoeris defense systems can use a base-exchange reaction to produce immune signaling molecules in response to phage infection. Imidazole is unlikely to be available in bacterial cells, but derivatives such as aminoimidazole ribonucleotide, carboxyaminoimidazole ribonucleotide, and aminoimidazole carboxamide ribonucleotide are intermediates in purine biosynthesis (*66*). It is possible that the free bases of these nucleotides could serve as substrates in ThsB-catalyzed base-exchange reactions, although we could not detect any new products when EcThsB1 was incubated with NAD^+^ and these bases (fig. S7, A and B). Histidine and its catabolites (e.g., imidazole acetate, histamine, urocanate, and imidazole propionate) (*67*, *68*) also contain an imidazole ring and could therefore be potential ThsB substrates. When this paper was under review, another group showed in a preprint that the BaThs system produces histidine adenine dinucleotide (also called His-ADPR) in response to phage infection and activates ThsA by binding to its macro domain (*69*). The single ThsB protein of BaThs has a tyrosine containing active site and a predicted structure (UniProt/AlphaFold Database: A0A806QQ11) similar to EcThsB2 and ThsB subfamily 9 members (Fig. 5), suggesting that this class of ThsB proteins produces histidine adenine dinucleotide as an immune signaling molecule. Intriguingly, EcThsB1^TIR^ could use the histidine catabolite histamine as a substrate in base-exchange reactions, but not histidine (fig. S7, A and D), and it is thus possible that EcThsB1 produces an adenine dinucleotide with a different imidazole-containing base to activate ThsA, or that additional domains/regions of EcThsB1 are required to form an active site capable of histidine adenine dinucleotide production.

In *Bacillus* Thoeris systems with multiple TIR-containing ThsB proteins, each ThsB protein most likely recognizes a different phage infection marker and initiates Thoeris defense independently of each other (*11*). We speculate that this is also true for the *E. coli* Thoeris system, but further studies will be required to determine the exact identity of cellular EcThsB1 and EcThsB2 substrates and signaling nucleotides, and whether both proteins can activate EcThsA upon phage infection.

Self-association is critical for the NADase activity of TIR domains (*36*, *37*, *58*), and they are often autoregulated by limiting their ability to oligomerize. The proneurodegenerative and octameric enzyme SARM1, for example, is autoinhibited by spatially separating the TIR domains from each other via binding to its ARM domains (*58*, *70*), while TIR domain–containing CBASS effectors such as stimulator of interferon genes–TIR (STING-TIR) and SMODS-associated and fused to various effector domains–TIR (SAVED-TIR) only oligomerize upon recognition of nucleotide signals (*9*, *71*). Our structural data suggest that EcThsB2 may be autoregulated, in this case, by a short C-terminal extension that binds to a surface region shown to be important for TIR domain self-association and active site formation in SARM1 and AbTir (*39*, *58*). AlphaFold2 modeling of the ThsB family (Fig. 5) shows that many ThsB proteins feature N- or C-terminal extensions or loop insertions that likely interfere with TIR domain assembly formation; we speculate that ThsB proteins have evolved these additional features to both interact with phage or phage-modified host components and to prevent TIR domain self-association in noninfected cells. In conclusion, our study reveals distinct immune signaling molecule requirements for SIR2-SLOG and TM-macro ThsA Thoeris systems and informs on the structure and mechanism of action by TM-macro ThsA effectors.

## MATERIALS AND METHODS

### Cloning

The EcThsA (GenBank: AAN80859.1), PcThsA (GenBank: WP_175361674.1), EcThsB1 (GenBank: AAN80857.1), and EcThsB2 (GenBank: AAN80858.1) genes were synthesized as gBlocks (Integrated DNA Technologies). EcThsA^Macro^ (residues 62 to 278), PcThsA^Macro^ (residues 63 to 278), EcThsB1^TIR^ (residues 165 to 307), and EcThsB2 (residues 1 to 169) were amplified by polymerase chain reaction and cloned into the pMCSG7 vector using ligation-independent cloning (LIC) (*72*). The resulting constructs were verified by sequencing.

### Site-directed mutagenesis

The EcThsA^Macro^ N114A and D100A mutants were produced using Q5 Site-Directed Mutagenesis (New England BioLabs), while the EcThsA^Macro^ DSTK and RQIRKF mutants and the EcThsB2 mutants were synthesized (gBlocks, Integrated DNA Technologies) and cloned into the pMCSG7 vector using LIC. Pure plasmids were prepared using the QIAprep Spin Miniprep Kit (Qiagen), and the sequences were confirmed by the Australian Genome Research Facility.

### Protein production

EcThsA^Macro^, PcThsA^Macro^, EcThsB1^TIR^, and EcThsB2 in the pMCSG7 vector [N-terminal His_6_-tag, tobacco etch virus (TEV) protease cleavage site] were produced in *E. coli* BL21 (DE3) cells, using the autoinduction method (*73*), and purified to homogeneity, using a combination of immobilized metal affinity chromatography (IMAC) and SEC. The cells were grown at 37°C, until an optical density at 600 nm of 0.6 to 0.8 was reached. The temperature was then reduced to 20°C, and the cells were grown overnight for approximately 16 hours. The cells were harvested by centrifugation at 5000*g* at 4°C for 15 min and stored at −80°C until used for purification.

### Protein purification

EcThsA^Macro^, PcThsA^Macro^, and EcThsB1^TIR^: The cell pellets were resuspended in 2 to 3 ml of lysis buffer [50 mM Hepes (pH 8.0), 500 mM NaCl, and 30 mM imidazole] per gram of cells. The resuspended cells were lysed using a sonicator and clarified by centrifugation (15,000*g* for 30 min). The clarified lysate was applied to a nickel HisTrap column (Cytiva) pre-equilibrated with 10 column volumes (CVs) of the wash buffer [50 mM Hepes (pH 8.0), 500 mM NaCl, and 30 mM imidazole] at a rate of 5 ml/min. The column was washed with 10 CVs of the wash buffer, followed by elution of bound proteins using elution buffer [50 mM Hepes (pH 8), 500 mM NaCl, and 250 mM imidazole]. The elution fractions were analyzed by SDS-PAGE, and the fractions containing the protein of interest were pooled and further purified on a S75 HiLoad 26/600 column pre-equilibrated with gel filtration buffer [10 mM Hepes (pH 7.5) and 150 mM NaCl]. The peak fractions were analyzed by SDS-PAGE, and the fractions containing EcThsA^Macro^, PcThsA^Macro^, and EcThsB1^TIR^ were pooled and concentrated to final concentrations of approximately 30 mg/ml (EcThsA^Macro^), 28 mg/ml (PcThsA^Macro^), and 2 mg/ml (EcThsB1^TIR^), flash-frozen as 10-μl aliquots in liquid nitrogen, and stored at −80°C.

EcThsB2: The cell pellets were resuspended in 2 to 3 ml of lysis buffer [50 mM Hepes (pH 8.0), 500 mM NaCl, and 30 mM imidazole] per gram of cells. The resuspended cells were lysed using a sonicator and clarified by centrifugation (15,000*g* for 30 min). The clarified lysate was applied to a nickel HisTrap column (Cytiva) pre-equilibrated with 10 CVs of the wash buffer [50 mM Hepes (pH 8.0), 500 mM NaCl, and 30 mM imidazole] at a rate of 5 ml/min. The column was washed with 10 CVs of the wash buffer, followed by elution of bound proteins using elution buffer [50 mM Hepes (pH 8), 500 mM NaCl, and 250 mM imidazole]. The elution fractions were analyzed by SDS-PAGE, and the fractions containing EcThsB2 were pooled, supplemented with TEV protease, and dialyzed into gel filtration buffer [10 mM Hepes (pH 7.5) and 300 mM NaCl] for 16 to 20 hours. After dialysis, cleaved EcThsB2 was reloaded onto the HisTrap column to remove the TEV protease, His_6_-tag, and contaminants. After the second IMAC step, EcThsB2 was further purified on a S75 HiLoad 26/600 column pre-equilibrated with gel filtration buffer. The peak fractions were analyzed by SDS-PAGE, and the fractions containing EcThsB2 were pooled and concentrated to a final concentration of approximately 33 mg/ml, flash-frozen as 10-μl aliquots in liquid nitrogen, and stored at −80°C.

### Crystallization and crystal structure determination

EcThsA: EcThsA^Macro^ crystals were produced using the hanging drop method, with drops containing 1 μl of protein (6.5 to 13.5 mg/ml) and 1 μl of well solution [14 to 24% (w/v) polyethylene glycol 1000 (PEG 1000), 0.2 M lithium sulfate, and 0.1 M phosphate-citrate buffer (pH 3.8 to 4.2)]. The crystals appeared within 1 to 5 days. Native or ADPR-soaked (1 hour, 10 mM final concentration in well solution) crystals were cryoprotected in glycerol [80% well solution and 20% (v/v) glycerol] before flash-cooling in liquid nitrogen. X-ray diffraction data were collected from a single crystal at the Australian Synchrotron MX2 beamline, using a wavelength of 0.9537 Å. Datasets were indexed and integrated using XDS (*74*) and scaled with AIMLESS within the Collaborative Computational Project No. 4 (CCP4) suite (*75*). Molecular replacement was initially attempted using several published macro domain structures as templates, but a solution could not be obtained. We determined the EcThsA^Macro^ structure by the iodide-SIRAS phasing method using the CRANK2 (*76*) pipeline of the CCP4 suite. Halide derivatization of EcThsA crystals was obtained by soaking native crystals for 10 min in cryoprotectant solution containing 500 mM NaI before flash-cooling in liquid nitrogen. X-ray diffraction data were collected from a single crystal using CuKα radiation (wavelength of 1.54 Å) generated by a Rigaku Micro-Max007 rotating anode x-ray generator operated at 40 kV and 30 mA. Diffraction images were recorded using a Pilatus 200K detector. Twelve iodine atoms were located by SHELXD (*77*), and automatic model building was performed using Buccaneer (*78*) and Refmac (*79*). The higher-resolution ligand-free and ADPR-complex structures were subsequently solved by molecular replacement using PHASER (*80*). The models were refined using Phenix (*81*), with iterative model building carried out between rounds of refinement using Coot (*82*). Structure validation was performed using MolProbity (*83*). Data processing and refinement statistics are provided in table S1. The coordinates and structure factors have been deposited in the PDB with IDs 8V6Q and 8V6R for the ligand-free and ADPR-bound structures, respectively.

PcThsA^Macro^: PcThsA^Macro^ crystals were produced using the hanging drop method, with drops containing 1 μl of protein (14 mg/ml) and 1 μl of well solution [14 to 24% (w/v) PEG 6000 0.2 M magnesium chloride and 0.1 M sodium acetate buffer (pH 5.0)]. The crystals appeared within 1 to 5 days. Crystals were cryoprotected in glycerol [80% well solution and 20% (v/v) glycerol] before flash-cooling in liquid nitrogen. X-ray diffraction data were collected from a single crystal at the Australian Synchrotron MX2 beamline, using a wavelength of 0.9537 Å. The structure was solved by molecular replacement using Phaser (*80*) and an AlphaFold2 model of PcThsA as a template (*84*). The model was refined and built using Phenix (*81*) and Coot (*82*), and structure validation was performed using MolProbity (*83*). Data processing and refinement statistics are provided in table S1. The coordinates and structure factors have been deposited in the PDB with ID 8V6S.

EcThsB2: EcThsB crystals were produced using the hanging drop method, with drops containing 1 μl of protein (15 mg/ml) and 1 μl of well solution [30% PEG Smear Broad, 10% ethylene glycol, 0.1 M potassium sodium tartrate tetrahydrate, and 0.1 M sodium cacodylate pH 5.5]. The crystals appeared within 1 to 5 days. We determined the EcThsB2 structure by the bromide-SAD phasing method using the SHELXC/D/E pipeline within the CCP4 suite (*85*). Halide derivatization of EcThsB crystals was obtained by soaking native crystals for 5 min in cryoprotectant solution (well solution with 30% ethylene glycol) containing 1 M KBr before flash-cooling in liquid nitrogen. Bromine atoms were located by SHELXD (*77*), and automatic model building was performed using Buccaneer (*78*) and Refmac (*79*). The model was refined and built using Phenix (*81*) and Coot (*82*). Structure validation was performed using MolProbity (*83*). Data processing and refinement statistics are provided in table S1. The coordinates and structure factors have been deposited in the PDB with ID 8V6T.

### Structure prediction of ThsA oligomers

Models of full-length (ligand-free) EcThsA and PcThsA dimers and a EcThsB1^TIR^ octamer were generated using AlphaFold2 multimer (*84*, *86*) implemented in the ColabFold interface (v1.5.2) available on the Google Colab platform using default settings (*87*). Models of full-length (ligand-free) EcThsA and PcThsA higher-order oligomers (octamers) were generated using AlphaFold2 multimer (*84*, *86*) implemented in the ColabFold interface (v1.5.1) using a local ColabFold installation with default settings. The confidence of AlphaFold2 models was evaluated by predicted local distance difference test (pLDDT) and predicted aligned error.

### Structure prediction and analysis of ThsB proteins

The sequences of 3257 ThsB proteins reported by Doron *et al.* (*3*) were obtained from the National Center for Biotechnology Information database. Redundancy was removed using the CD-HIT tool (*88*), which resulted in a set of 446 ThsB proteins with ≤90% sequence identity. The structures of these 446 ThsB proteins were predicted using AlphaFold2 (*84*) implemented in the ColabFold interface (v1.5.2) available on the Google Colab platform (*87*). For each protein, five models were generated, and the best model (ranked_0), determined by the average pLDDT score, was used for further analyses. Structures of ThsB proteins with truncated TIR domains were removed, which resulted in a final dataset of 426 AlphaFold2 predicted ThsB structures. A structure-based phylogenetic tree was constructed using Foldtree (*89*) on the Google Colab platform (https://colab.research.google.com/github/DessimozLab/fold_tree/blob/main/notebooks/Foldtree.ipynb). The Interactive Tree of Life (iTOL) tool was used for tree visualization and annotation (*90*).

### NMR-based NADase assay

NMR samples were prepared in 175-μl Hepes-buffered saline (HBS) buffer [50 mM Hepes and 150 mM NaCl (pH 7.5)], 20 μl D_2_O, and 5 μl of deuterated dimethyl sulfoxide (DMSO-d6), resulting in a total volume of 200 μl. Each sample was subsequently transferred to a 3-mm Bruker NMR tube. All ^1^H NMR spectra were acquired with a Bruker Avance III HDX 800 MHz spectrometer equipped with a ^1^H/^13^C/^15^N triple resonance cryoprobe at 298 K. To suppress resonance from H_2_O, a water suppression pulse program (P3919GP), using a 3-9-19 pulse sequence with gradients (*91*, *92*), was implemented to acquire spectra with an acquisition delay of 2 s and 32 scans per sample. All spectra were processed by TopSpin (Bruker) and Mnova 11 (Mestrelab Research) as per previous studies (*58*, *70*).

### STD NMR

Samples for STD NMR were prepared with the same solvents as for the NMR-based NADase assays, and spectra were also acquired with the same NMR spectrometer. The pulse sequence STDDIFFGP19.3, in-built within the TopSpin program (Bruker), was used to acquire STD NMR spectra (*93*). This pulse sequence consists of a 3-9-19 water suppression pulse, the parameters of which were obtained from the water suppression pulse program (P3919GP), to suppress the resonance from H_2_O. The on-resonance irradiation was set close to protein resonances at 0.8 parts per million (ppm), whereas the off-resonance irradiation was set far away from any protein or ligand resonances at 300 ppm. A relaxation delay of 4 s was used, out of which a saturation time of 3 s was used to irradiate the protein with a train of 50-ms Gaussian shaped pulses. The number of scans was 256. All STD NMR spectra were processed by TopSpin (Bruker) and Mnova 11 (Mestrelab Research).

### Isothermal titration calorimetry

ITC experiments were performed in duplicate on Nano ITC (TA Instruments). All proteins and compounds were dissolved in a buffer containing 10 mM Hepes (pH 7.5) and 150 mM NaCl. The baseline was equilibrated for 600 s before the first injection. A total of 0.3 mM ADPR, NAD^+^, 2′cADPR, and 3′cADPR were titrated as 30 injections of 1.44 μl every 200 s into 40 μM RQIRKF EcThsA^Macro^ mutant. IAD (0.5 mM) was titrated as 30 injections of 1.44 μl every 200 s into 44 μM RQIRKF EcThsA^Macro^ mutant. The heat change was recorded by injection over time, and the binding isotherms were generated as a function of molar ratio of the protein solution. The *K*_d_ values were obtained after fitting the integrated and normalized data to a single-site binding model using NanoAnalyze (TA Instruments).

### Size exclusion chromatography–coupled multiangle light scattering

A DAWN HELEOS II 10-angle light-scattering detector coupled with an Optilab rEX refractive index detector (Wyatt Technology), combined with a Superdex 200 5/150 Increase size exclusion column (Cytiva), connected to a Prominence HPLC (Shimadzu), was used for SEC-MALS. The column was equilibrated in gel filtration buffer, and 30 μl of EcThsA^Macro^ was run through the column at 0.25 ml/min. Molecular masses were calculated using Astra 6.1 (Wyatt Technology).

### Negative-stain EM

EcThsA^Macro^ (at 1 mg/ml) was incubated with 1 mM ADPR for 1 hour at 4°C. EcThsB1^TIR^ (at 2 mg/ml) was incubated with 2 mM **3AD** at 25°C for 1 hour. Four microliters of sample was placed on a carbon-coated copper grid and incubated for 60 s. The grid was then washed with Milli-Q H2O and stained with 1% uranyl acetate for 60 s and air-dried. The images were collected on a JEOL JEM-1011 TEM 120-kV transmission electron microscope at ×25,000 magnification at 120 keV.

### Production and purification of IAD

Production reactions for IAD were performed using conditions similar to the ^1^H NMR NADase assay. A solvent volume of 4 ml was used for each reaction, consisting of 98% HBS buffer [50 mM Hepes and 150 mM NaCl (pH 7.5)] and 2% (v/v) DMSO. For IAD production, 0.5 μM EcThsB1^TIR^, 5 mM imidazole, and 5 mM NAD^+^ were added to the solution. These reactions were performed at room temperature and monitored intermittently by ^1^H NMR. To stop the reactions, the His_6_-tagged protein was removed by incubating the mixture with 200 μl of HisPur nickel–nitrilotriacetic acid resin for 30 to 60 min. The resin was subsequently removed by centrifugation at 1500*g* for 1 min, and the supernatant was subjected to HPLC-based separation to purify the base-exchange products. A Shimadzu Prominence HPLC equipped with a Synergi 4-μm Hydro-RP 80-Å column was used for separation. The mobile phase consisted of phase A [0.05% (v/v) formic acid in water] and phase B [0.05% (v/v) formic acid in methanol]. Different gradients, flow rates, and run times were applied depending on prior optimization with individual reaction mixtures. Product peaks were confirmed by comparison with individual chromatograms of NAD^+^, nicotinamide (NAM), ADPR, and imidazole. Fractions corresponding to the product peaks were collected, concentrated, and lyophilized and stored at −20°C.

### Characterization of IAD

^1^H-NMR (800 MHz, D_2_O): δ 8.91 (s, 1H), 8.56 (s, 1H), 8.35 (s, 1H), 7.62 (br, 1H), 7.44 (br, 1H), 6.09 (d, *J* = 5.5 Hz, 1H), 5.81 (d, *J* = 5.1 Hz, 1H), 4.71 (br, 1H), 4.46 (dd, *J* = 5.1, 3.9 Hz 1H), 4.42 (t, *J* = 5.0 Hz, 1H), 4.32 (br, 1H), 4.30 (br, 1H), 4.09 to 4.18 (m, 4H); ^13^C-NMR (200 MHz, D_2_O): δ 150.2, 148.5, 145.0, 142.4, 133.6, 120.4, 119.1, 118.6, 91.7, 87.8, 84.8, 84.2, 76.0, 74.5, 70.3, 70.2, 65.1, 64.8; high-resolution MS [mass/charge ratio (*m/z*)]: [M-H]^−^ calculated for C_18_H_24_N_7_O_13_P_2_, 608.0913; found, 608.0899. Purity > 90% (^1^H-NMR).

### Liquid chromatography–mass spectrometry

Thirty microliters of PcThsA (1050 μM) was added to 90 μl of ice cold 100% methanol (MeOH) and left at −20°C overnight. Protein precipitant was removed through centrifugation, the supernatant was transferred to a new 1.5-ml Eppendorf tube, and a MiVac Quattro Concentrator was used to evaporate MeOH. The pellet was resuspended in 25 μl of Milli-Q H_2_O. Eight microliters was injected for LC-MS analysis on a Thermo Fisher Scientific UltiMate 3000 HPLC interfaced with Bruker micrOTOF-Q II system (Bruker Corporation, Massachusetts, USA). A Synergi Hydro-RP 4 μ 80 Å was used for LC separation at 40°C. The column was equilibrated with 100% mobile phase A (0.2% formic acid in water). Mobile phase B was 0.2% formic acid in 80% acetonitrile. The gradient was as follows: 0 to 3 min 100 % A (system was diverted to waste for the first 4 min), 3 to 7 min increased to 40 % B, 7 to 8.5 held at 40 %, 8.2 to 9 min decreased to 0%, and 9 to 11 min held to 0 % B; 0 to 7 min 100 % A, 7 to 10 min 30% B, 10 to 12 min 30% B, 12 to 14 min 0% B, and 14 to 16 min 0% B; 300 μl/min. An ESI MS scan *m/z* 50 to 3000 in negative ion mode was performed. The source parameters were as follows: nebulizer gas, 0.6; dry gas, 8 liters/min; dry temperature, 250°C; end plate offset, −550 V; capillary, +3100 V; end plate offset, −550 V; capillary +3100 V; in-source collision induced dissociation, 0.0 eV; hexapole radio frequency, 250 Vpp; and collision RF, 150 Vpp. Acquisition was controlled using Hystar3.2 SR2, MicroTof Control 1.3, and Chromeleon 6.8. Data were analyzed using Data Analysis 4.0.

## Supplementary Materials

### This PDF file includes:

Figs. S1 to S12 Table S1 Legend for table S2 References
