# Supplementary material for: Structural characterization of macro domain–containing Thoeris antiphage defense systems
Source: Sci Adv. 2024 Jun 26;10(26):eadn3310. doi: 10.1126/sciadv.adn3310 (PMC11204291; doi:10.1126/sciadv.adn3310)
Supplement: Supplementary file 1 — Figs. S1 to S12 Table S1 Legend for table S2 References [file sciadv.adn3310_sm.pdf]

Science Advances
AAAS

Supplementary Materials for

Structural characterization of macro domain–containing Thoeris antiphage defense systems

Yun Shi *et al.*

Corresponding author: Thomas Ve, t.ve@griffith.edu.au

Sci. Adv. **10**, eadn3310 (2024)
DOI: 10.1126/sciadv.adn3310

The PDF file includes:

Figs. S1 to S12
Table S1
Legend for table S2
References

Other Supplementary Material for this manuscript includes the following:

Table S2

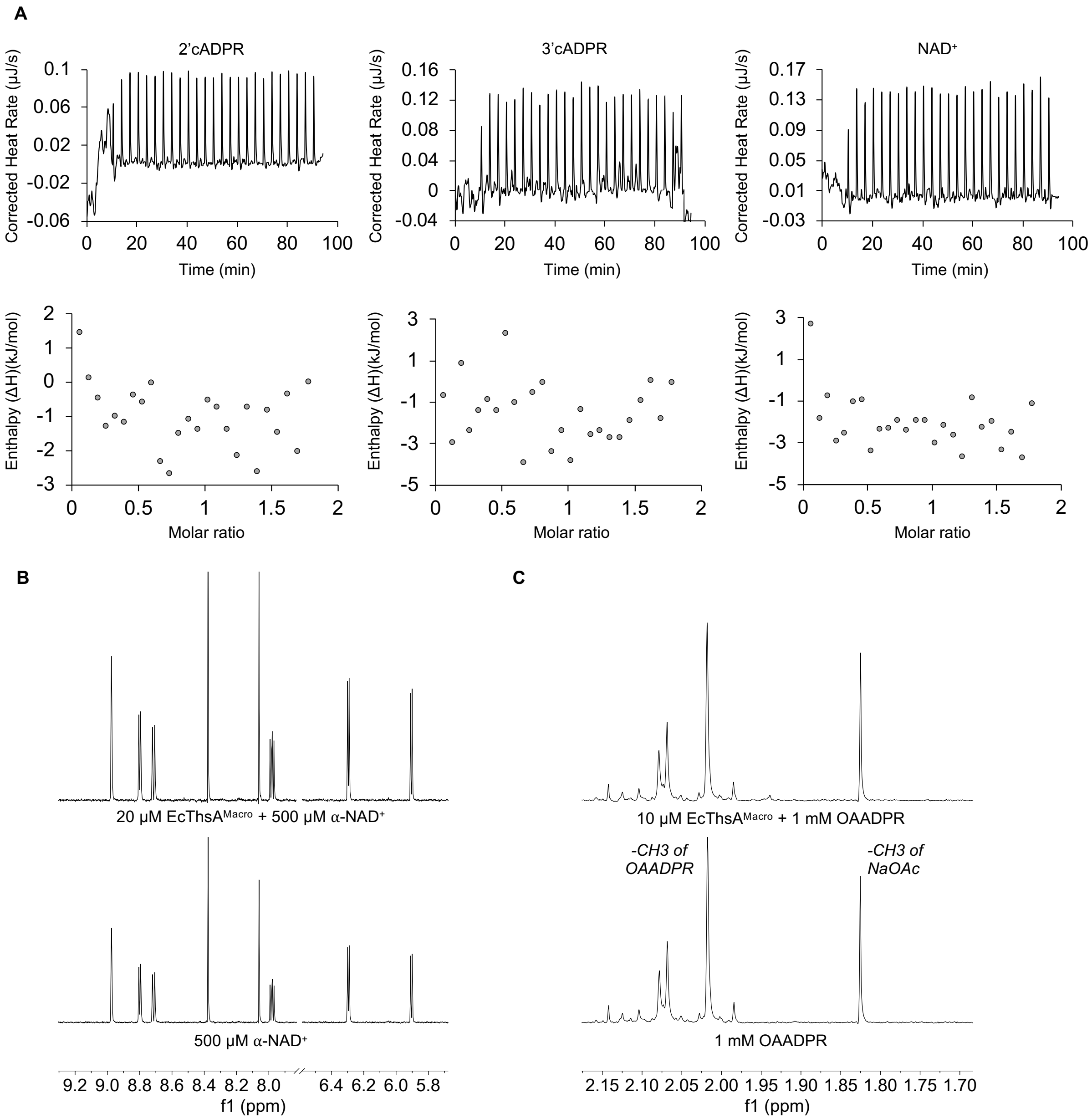

Fig. S1. ITC and NMR analyses of EcThsA^{Macro}. (A) Raw (top panel) and integrated (bottom panel) ITC data for the titration of 0.3 mM 2'cADPR (left), 3'cADPR (middle), and NAD^{+} (right) with 40 µM EcThsA^{Macro}. The RQIRKF EcThsA^{Macro} mutant, which does not aggregate in the presence of ADPR, was used for the ITC experiments. (B-C) Expansions of ^{1}H NMR spectra showing the absence of activity for EcThsA^{Macro} incubated with either α-NAD^{+} (B) or OAADPR (C). Incubation time was 16 h for all spectra.

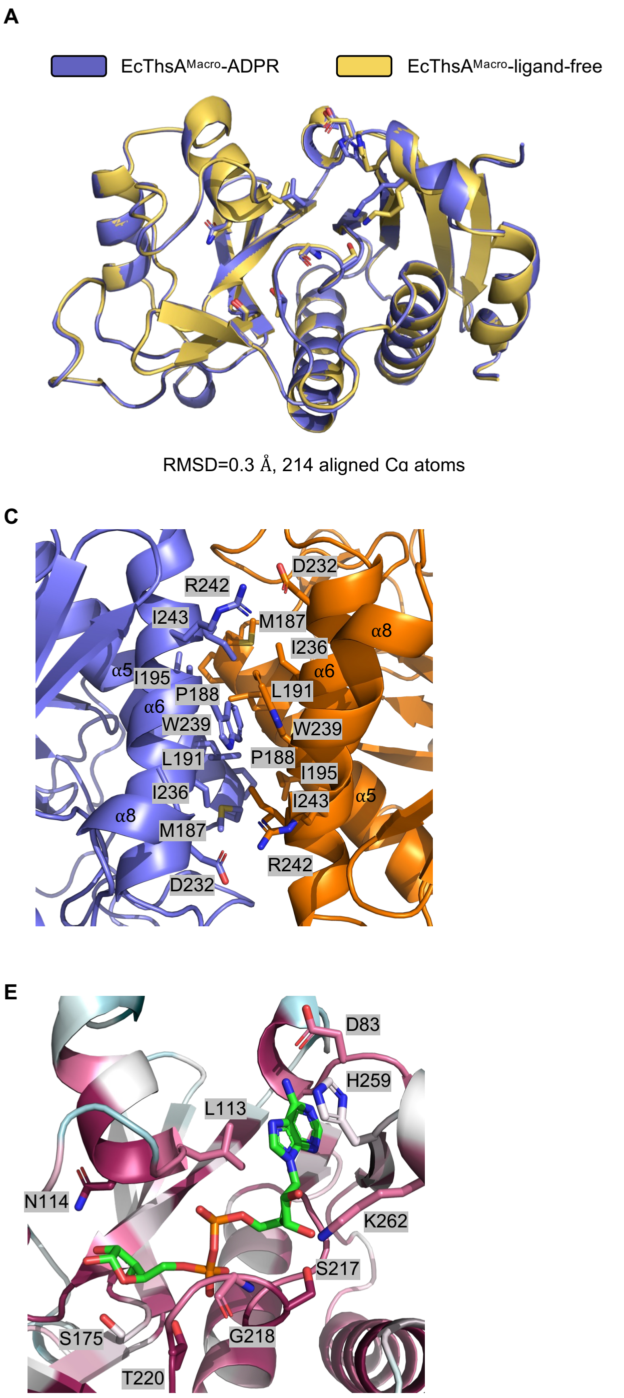

Fig. S2. Structural analyses of EcThsA^{Macro}. (A) Structural superpositions (Cα atoms) of the EcThsA^{Macro}-ADPR complex with the ligand-free structure. (B) Cartoon representation of EcThsA^{Macro} (slate) with the unique α_3-β_4 loop and β_6-β_7 hairpin highlighted in green and orange, respectively. Residues stabilizing the α_3-β_4 loop conformation (D100, T101, D130, R150 and K154) are shown in stick representation. (C) Enlarged cutaway of the dimer interface in the EcThsA^{Macro} crystal structure with selected interface residues highlighted in stick representation. (D) EcThsA^{Macro} monomer (surface representation) colored by sequence conservation. Cyan

corresponds to variable regions, while purple corresponds to conserved regions. Sequence conservation was calculated by ConSurf (*94*). (E) Enlarged cutaway of the ADPR binding pocket (cartoon representation) in $EcThsA^{Macro}$ colored by sequence conservation.

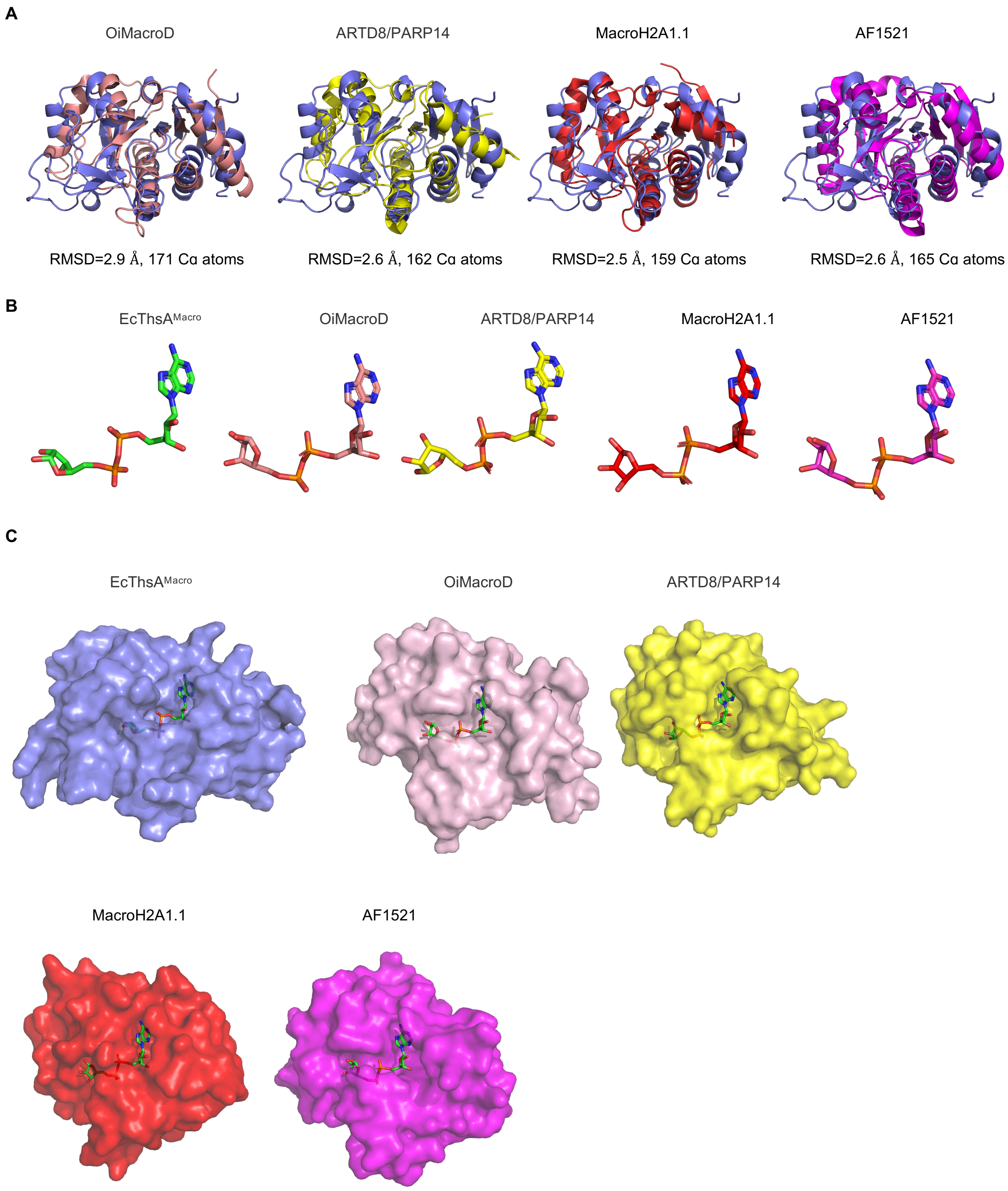

Fig. S3. Structural comparisons of macro domains. (A) Structural superpositions (Cα atoms) of EcThsA^{Macro} (slate) with the macrodomains of *Oceanobacillus Iheyensis* OiMacroD (pink; PDB: 5L9K), ARTD8/PARP14 (yellow; PDB: 3VFQ) MacroH2A1.1 (red; PDB: 3IID) and *Archaeoglobus fulgidus* AF1521 (magenta; PDB: 2BFQ) (*45, 55-57*). (B) Binding mode of ADPR

(stick representation) in superimposed structures in (A). Green: $EcThsA^{Macro}$; pink: OiMacroD; red: MacroH2A1.1; yellow: ARTD8/PARP14; and magenta: Af1521. (C) Surface representation of $EcThsA^{Macro}$ (slate), OiMacroD (pink), ARTD8/PARP14 (yellow), MacroH2A1.1 (red) and Af1521 (purple). ADPR is shown in stick representation (green).

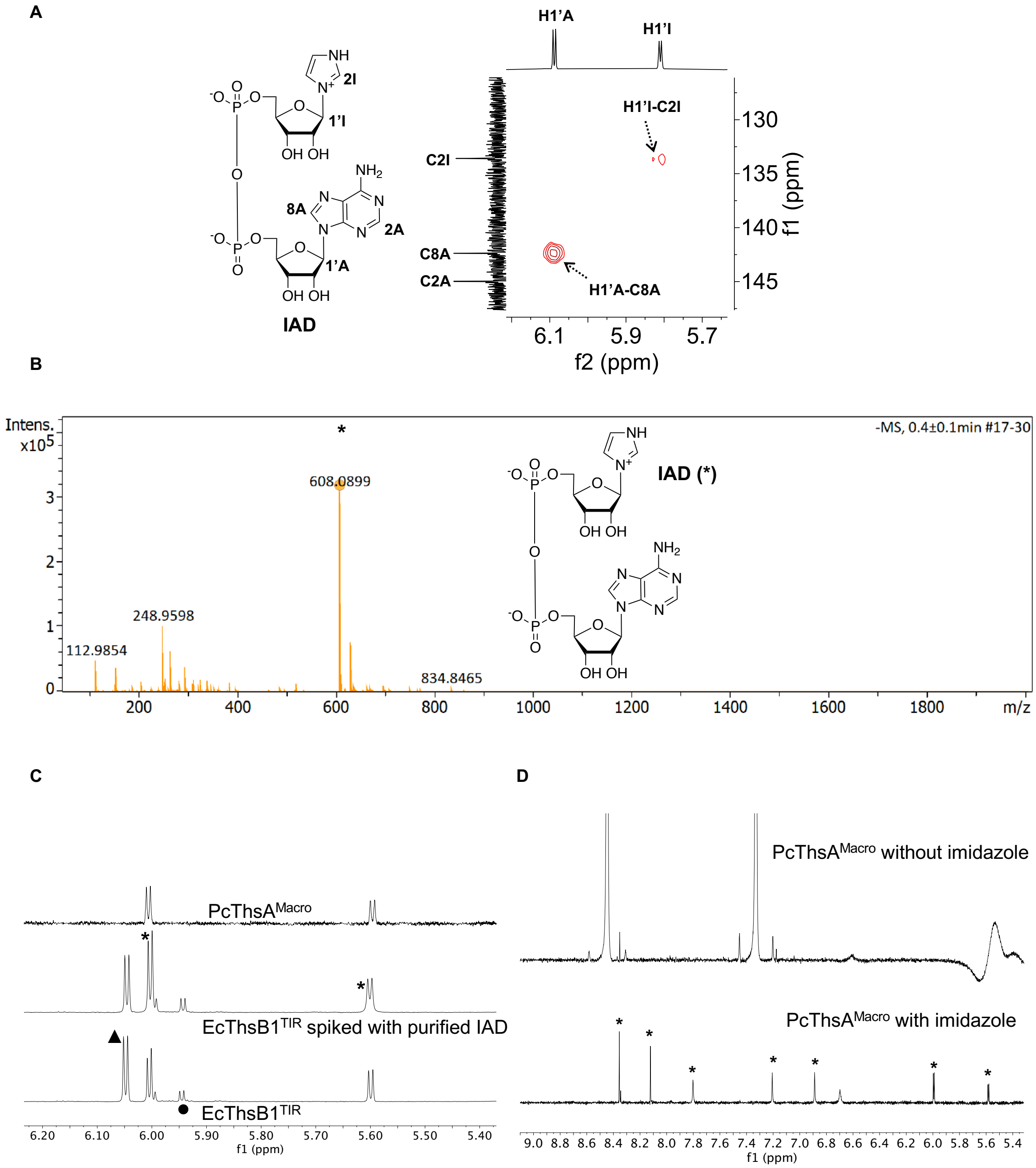

Fig. S4. MS and NMR analyses of IAD and PcThsA^{Macro} nucleotide extracts. (A) Chemical structure of IAD and expansion of the ^{1}H-^{13}C HMBC spectrum of a purified IAD sample, showing correlations through glycosidic linkages between imidazole and its adjacent ribose (H1'I-C2I) as well as between adenine and its adjacent ribose (H1'A-C8A). (B) High-resolution MS analysis of a purified IAD sample, showing the IAD (*) peak. (C) Expansions of ^{1}H NMR spectra showing the presence of IAD in PcThsA^{Macro} nucleotide extract. Bottom: a sample of 0.25 µM EcThsB1^{TIR} + 1 mM NAD^{+} + 1 mM imidazole incubated for 67 h. The peaks corresponding to NAD^{+} and ADPR are labelled by ● and ◆, respectively. Middle: the same sample spiked with 500 µM purified IAD to verify IAD peaks (*). Top: PcThsA^{Macro} nucleotide extract showing the same IAD

peaks as in the EcThsB1^{TIR} sample. (D) Expansions of ^{1}H NMR spectra of PcThsA^{Macro} nucleotide extracts. Top: nucleotide extract from PcThsA^{Macro} purified without imidazole in the lysis buffer. Bottom: nucleotide extract from PcThsA^{Macro} purified with 30 mM imidazole in the lysis buffer, with IAD peaks labelled (*).

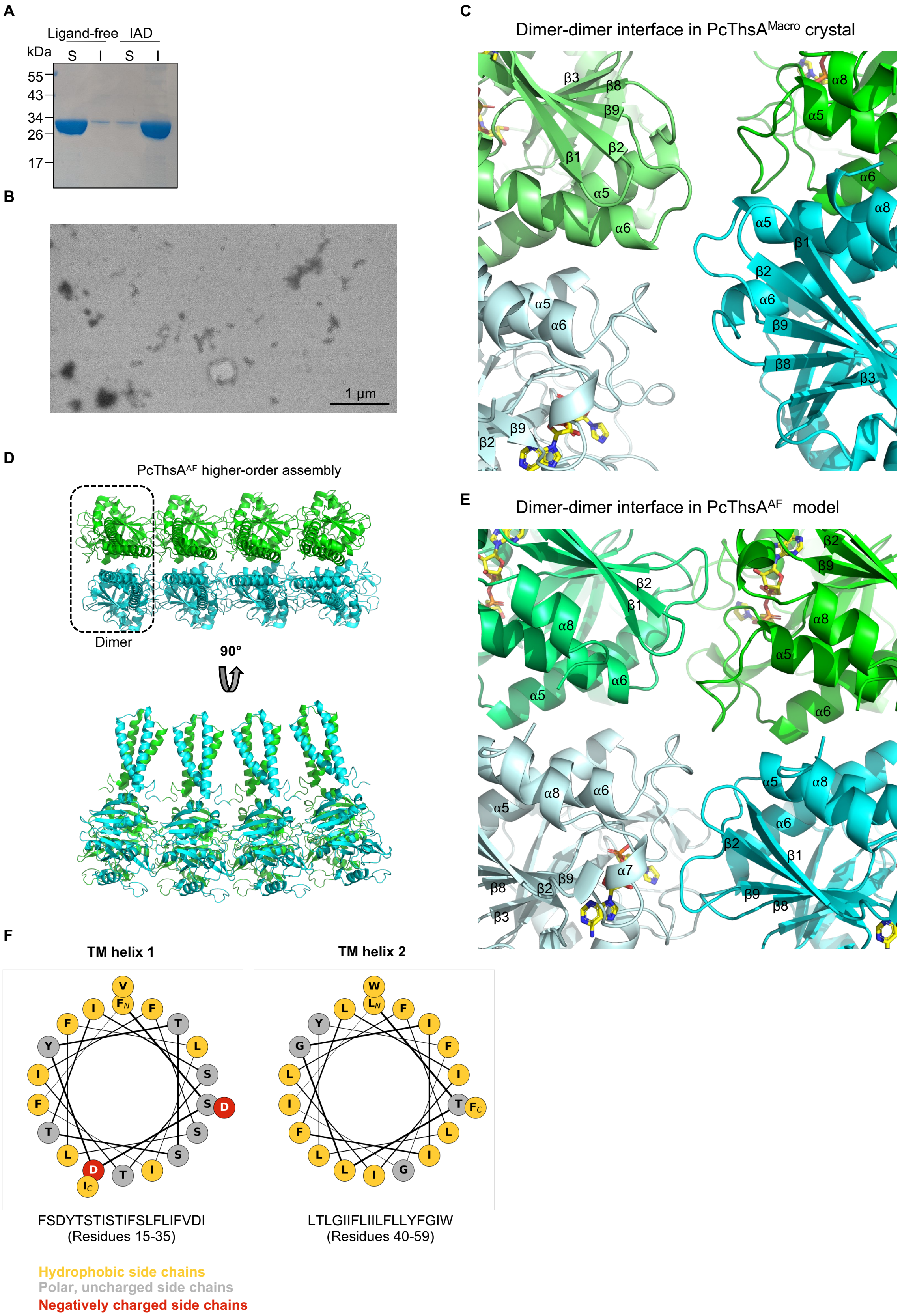
A
Ligand-free
IAD
kDa
S
I
S
I
55
43
34
26
17
B
1 μm
C
Dimer-dimer interface in PcThsA^Macro crystal
β3
β8
β9
β1
β2
α5
α6
α8
β3
D
PcThsA^AF higher-order assembly
Dimer
90°
E
Dimer-dimer interface in PcThsA^AF model
α7
F
TM helix 1
TM helix 2
FSDYTSTISTIFSLFLIFVDI
(Residues 15-35)
LTLGIIFLIILFLLYFGIW
(Residues 40-59)
Hydrophobic side chains
Polar, uncharged side chains
Negatively charged side chains

Fig. S5. Negative-stain EM and crystallographic analyses of ThsA. (A) SDS-PAGE analysis of the soluble (S) and insoluble (I) fractions after incubation of EcThsA^{Macro} (47 uM) with IAD at 4ºC for 1 h. The data are representative of three independent experiments (using the same protein batch). (B) Negative-stain EM image of EcThsA^{Macro} incubated with ADPR at 4ºC for 1 h. (C) Enlarged cutaways of the macro domain dimer-dimer interface in the PcThsA^{Macro} crystal structure. (D) Higher-order oligomer model of PcThsA predicted with AlphaFold2 Multimer (PcThsA^{AF}). (E) Enlarged cutaway of the macro domain dimer-dimer interface in the PcThsA^{AF} model. (F) Helical-wheel plots of the EcThsA TM domain.

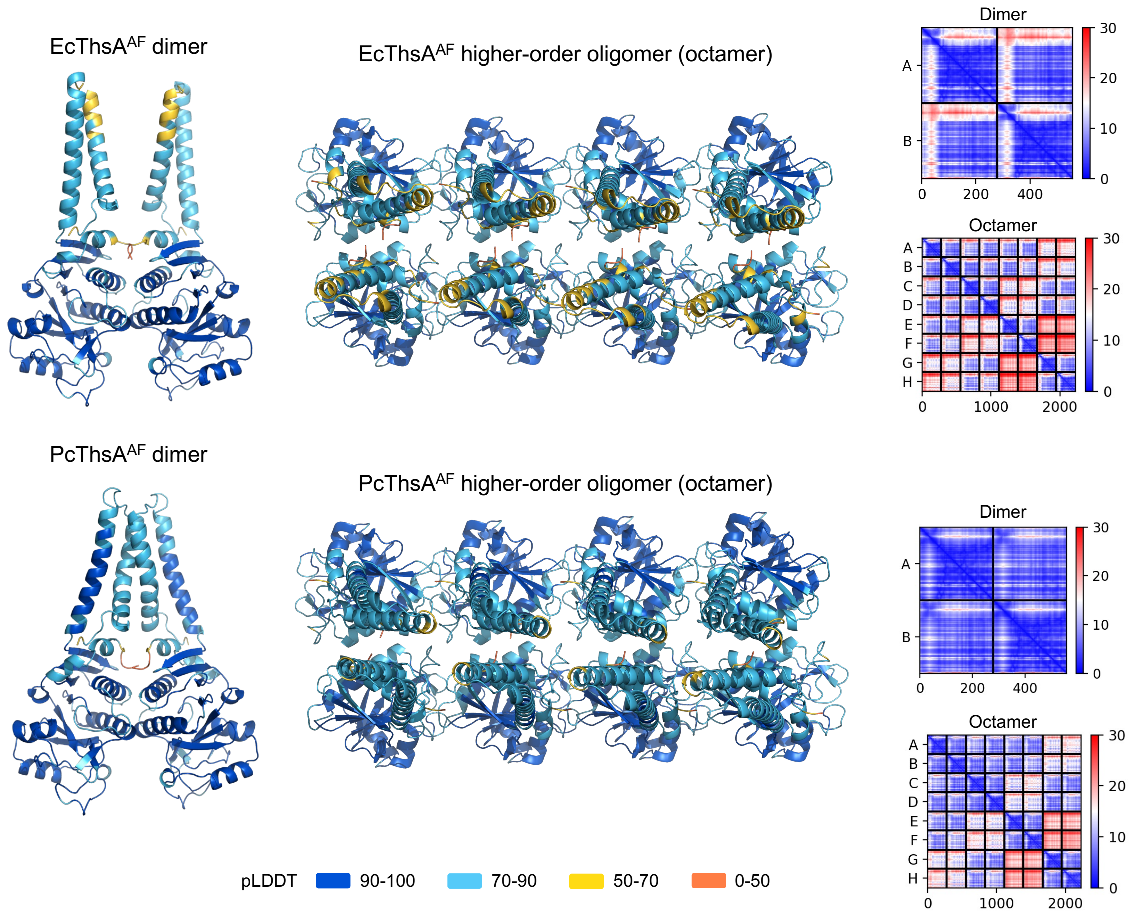

Fig. S6. AlphaFold2 models of EcThsA and PcThsA. EcThsA^{AF} and PcThsA^{AF} dimers and higher-order oligomers (octamers) colored by the predicted local distance difference test (pLDDT) score, with predicted aligned error (PAE) plots for the top-ranked models.

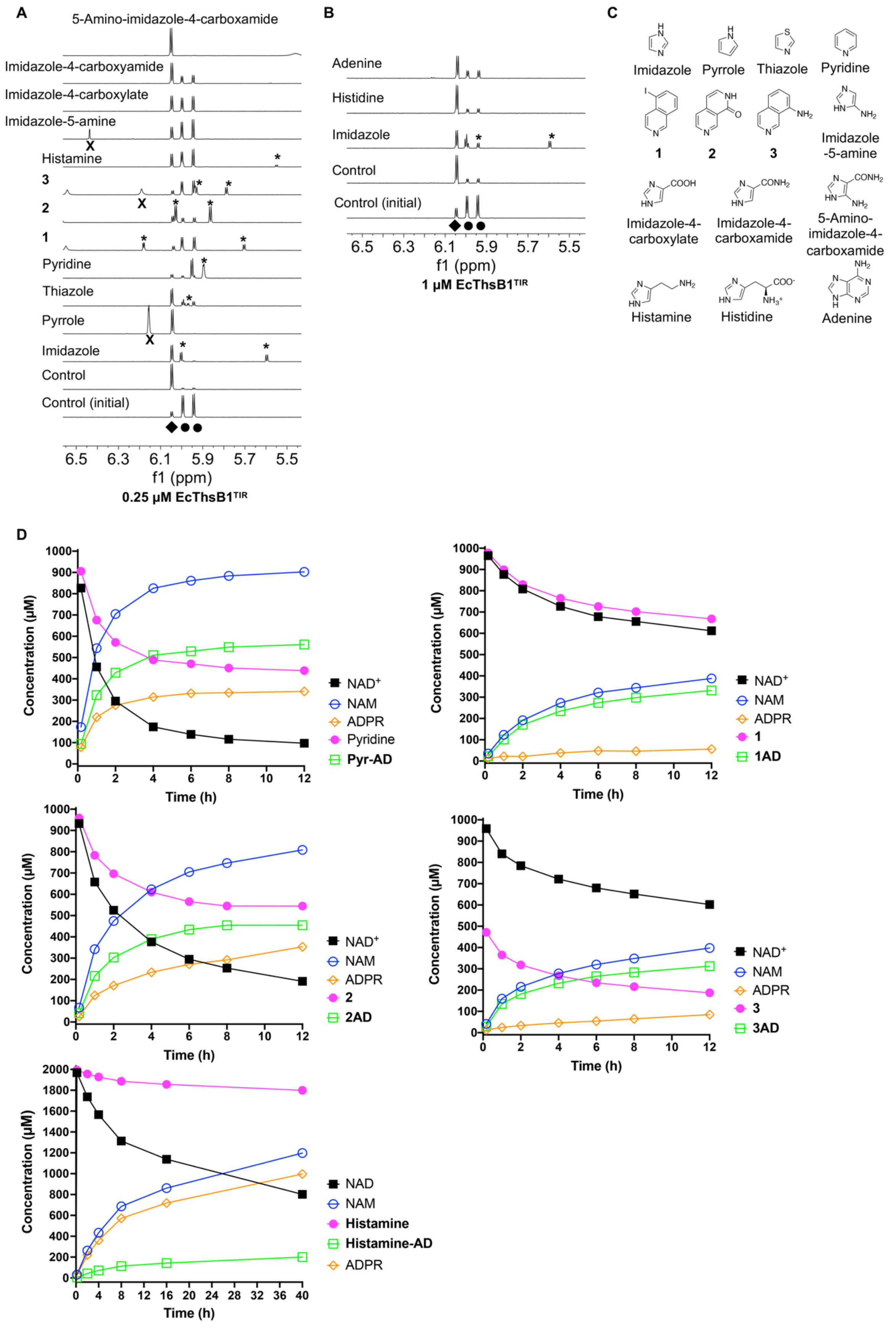
A
5-Amino-imidazole-4-carboxamide
Imidazole-4-carboxyamide
Imidazole-4-carboxylate
Imidazole-5-amine
Histamine
3
2
1
Pyridine
Thiazole
Pyrrole
Imidazole
Control
Control (initial)
6.5 6.3 6.1 5.9 5.7 5.5
f1 (ppm)
0.25 μM EcThsB1TIR
B
Adenine
Histidine
Imidazole
Control
Control (initial)
6.5 6.3 6.1 5.9 5.7 5.5
f1 (ppm)
1 μM EcThsB1TIR
C
Imidazole
Pyrrole
Thiazole
Pyridine
1
2
3
Imidazole -5-amine
Imidazole-4-carboxylate
Imidazole-4-carboxamide
5-Amino-imidazole-4-carboxamide
Histamine
Histidine
Adenine
D
Concentration (μM)
Time (h)
NAD+
NAM
ADPR
Pyridine
Pyr-AD
1
1AD
2
2AD
3
3AD
NAD
Histamine
Histamine-AD

Fig. S7. EcThsB1^{TIR} base-exchange reactions. (A-B) Expansions of ^{1}H NMR spectra showing the anomeric region (5.5 ~ 6.5 ppm) for various samples of EcThsB1^{TIR} + 1 mM NAD$^+$ + 1 mM heterocyclic base (except for EcThsB1^{TIR} + 2 mM NAD$^+$ + 2 mM Histamine). The incubation time was 12 h (A) and 1 h (B), respectively. The peaks corresponding to NAD$^+$, ADPR, and the heterocyclic base are labelled by ●, ◆, and **X**, respectively. New anomeric peaks are labelled by *, indicating the formation of base-exchange products from imidazole, thiazole (trace amount), pyridine, **1** (5-iodo-isoquinoline), **2** (1,2-Dihydro-2,7-naphthyridin-1-one), **3** (8-amino-isoquinoline), and histamine, respectively. (C) Chemical structures of the bases tested in (A-B). (D) Reaction progress curves for samples of 0.25 μM EcThsB1^{TIR} incubated with 1 mM NAD$^+$ + 1 mM pyridine (top, left), 1 mM NAD$^+$ + 1 mM **1** (top, right), 1 mM NAD$^+$ + 1 mM **2** (middle, left), 1 mM NAD$^+$ + 500 μM **3** (middle, right) and 2 mM NAD$^+$ + 2 mM Histamine (bottom, left), respectively.

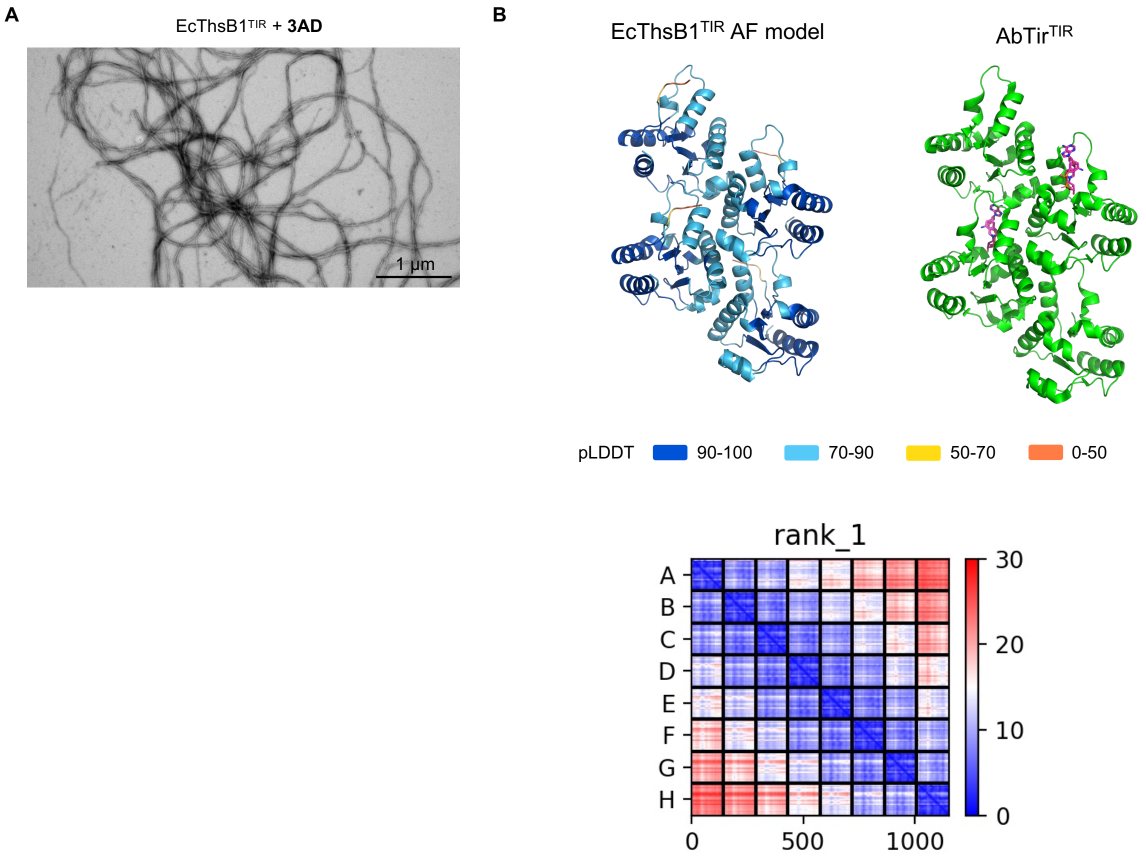

Fig. S8. Negative-stain EM and AlphaFold2 analyses of EcThsB1^{TIR}. (A) Negative-stain image of EcThsB1^{TIR}:**3AD** filaments. (B) AlphaFold2 model of an EcThsB1^{TIR} oligomer (colored by the pLDDT score, with PAE plot for the top-ranked model) and cryo-electron microscopy (cryoEM) structure of the *Acinetobacter baumannii* Tir TIR domain filament (AbTir^{TIR}; PDB: 7UXU). The structure of an EcThsB1^{TIR} octamer was predicted by AlphaFold2, but only the 4 central chains are displayed.

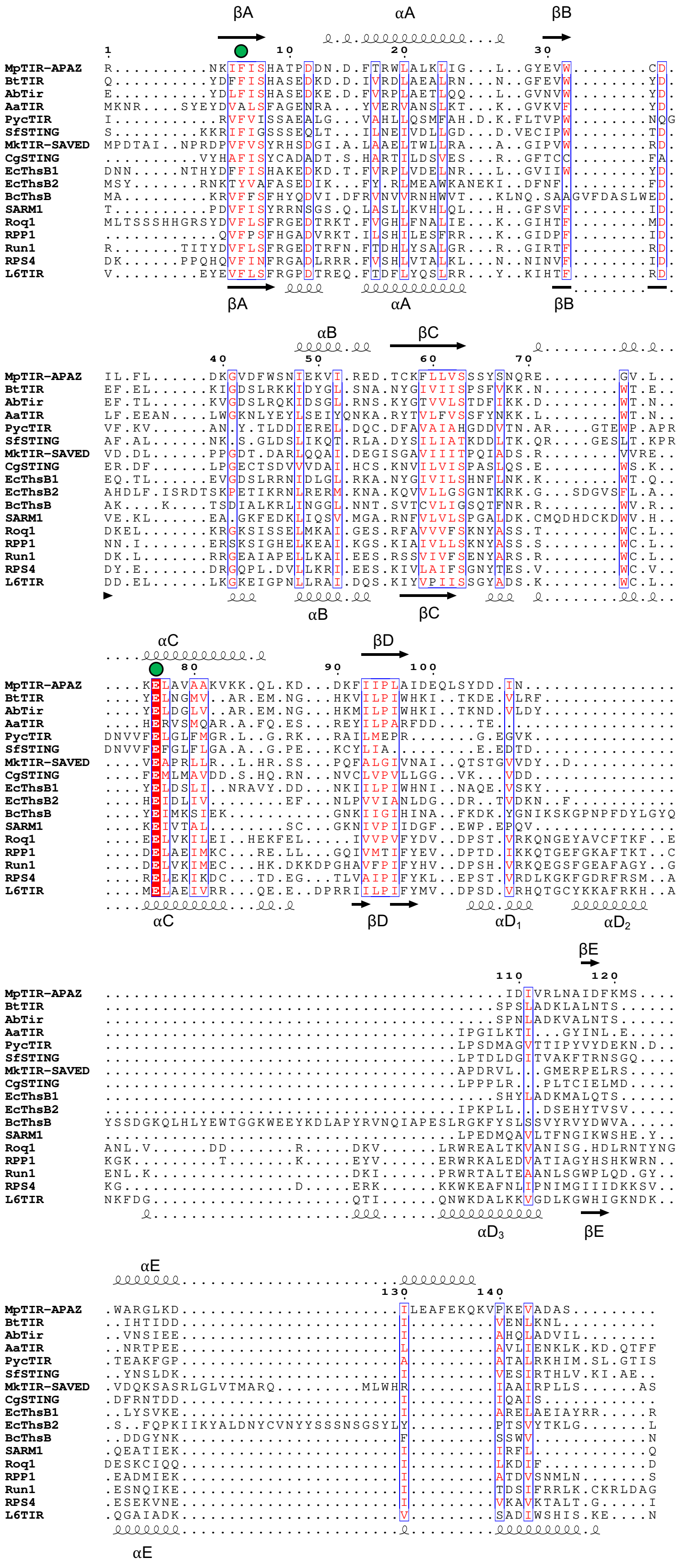

βA
αA
βB
αB
βC
αC
βD
αD1
αD2
βE
αD3
αE
MpTIR-APAZ
BtTIR
AbTir
AaTIR
PycTIR
SfSTING
MkTIR-SAVED
CgSTING
EcThsB1
EcThsB2
BcThsB
SARM1
Roq1
RPP1
Run1
RPS4
L6TIR

Fig. S9. Multiple sequence alignment of bacterial, plant, oyster, and human TIR domains with NADase activity. The alignment was formatted using ESPript (*97*). Green circles indicate selected active-site residues. Strictly conserved residues are indicated in white letters with a red box and similar residues are indicated in red letters with a blue frame. TIR domains from bacteria: MpTIR-APAZ (*Maribacter polysiphoniae*, residues 1-147, PDB: 8SPO) (*98*), BtTIR (*Bacteroides thetaiotaomicron*, residues 156-287, PDB: 7UXR), AbTir (*Acinetobacter baumannii*, residues 157-290, PDB: 7UXU) (*39*), AaTIR (*Aquimarina amphilecti*, residues 1-144), PycTIR (*Burkholderia cepacian*, residues 153-303), SfSTING (*Sphingobacterium faecium*, residues 1-145, PDB: 7UN8) (*71*), MkTIR-SAVED (*Microbacterium ketosireducens*, residues 1-164, PDB: 7QQK) (*9*), EcThsB1 (*E. coli*, residues 165-307), EcThsB2 (*E. coli*, residues 1-169), and BcThsB (*Bacillus cereus* MSX-D12, residues 1-192; PDB: 6LHY) (*51*); plants: Roq1 (*Nicotiana benthamiana*, residues 1-178, PDB: 7JLX) (*99*), RPP1 (*Arabidopsis thaliana*, residues 86-249, PDB: 7CRC) (*95*), Run1 (*Vitis rotundifolia*, residues 23-198, PDB: 6O0W) (*36*), RPS4 (*Arabidopsis thaliana*, residues 4-171, PDB: 4C6R) (*100*) and L6 (*Linum usitatissimum*, residues 34-204, PDB: 3OZI) (*101*); oyster: CgSTING (Crassostrea gigas residues 30-160, PDB: 6WT6) (*10*); and human SARM1 (residues 561-700, PDB: 6O0R) (*36*).

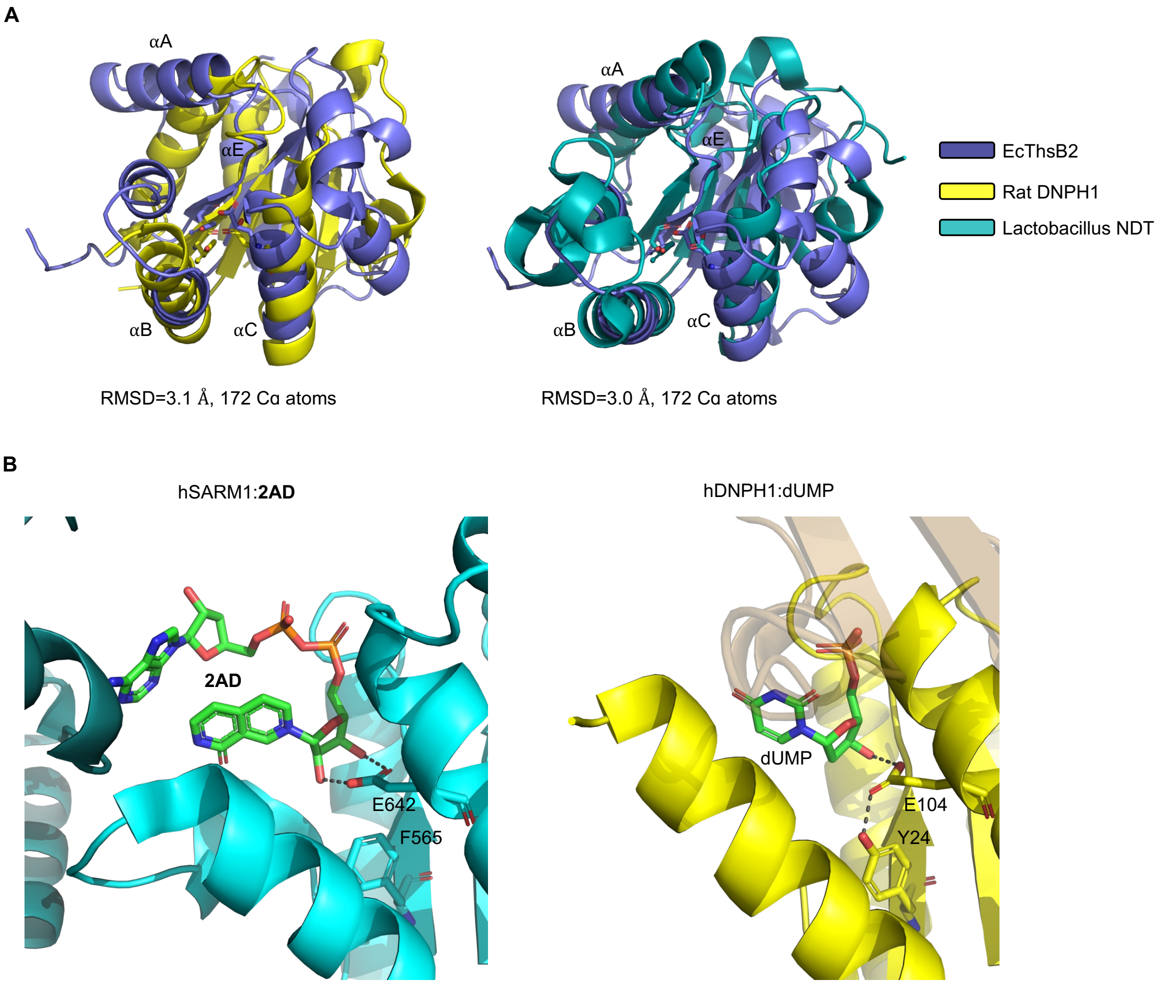

Fig. S10. Structural analyses of EcThsB2. (A) Structural superpositions (Cα atoms) of EcThsB2 (slate) with rat DNPH1 (PDB: 4FYH, yellow) (*96*) and *Lactobacillus* NDT (PDB: 1F8X, cyan) (*59*). (B) Ribbon representation of active-site regions in the SARM1:**2AD** (PDB: 7NAH) and human DNPH1:2'-deoxyuridine 5'-monophosphate (dUMP) (PDB: 8OSC) complexes (*58, 102*). Active-site tyrosine, phenylalanine and glutamate residues are shown in stick representation and hydrogens bonds are shown as grey dotted lines.

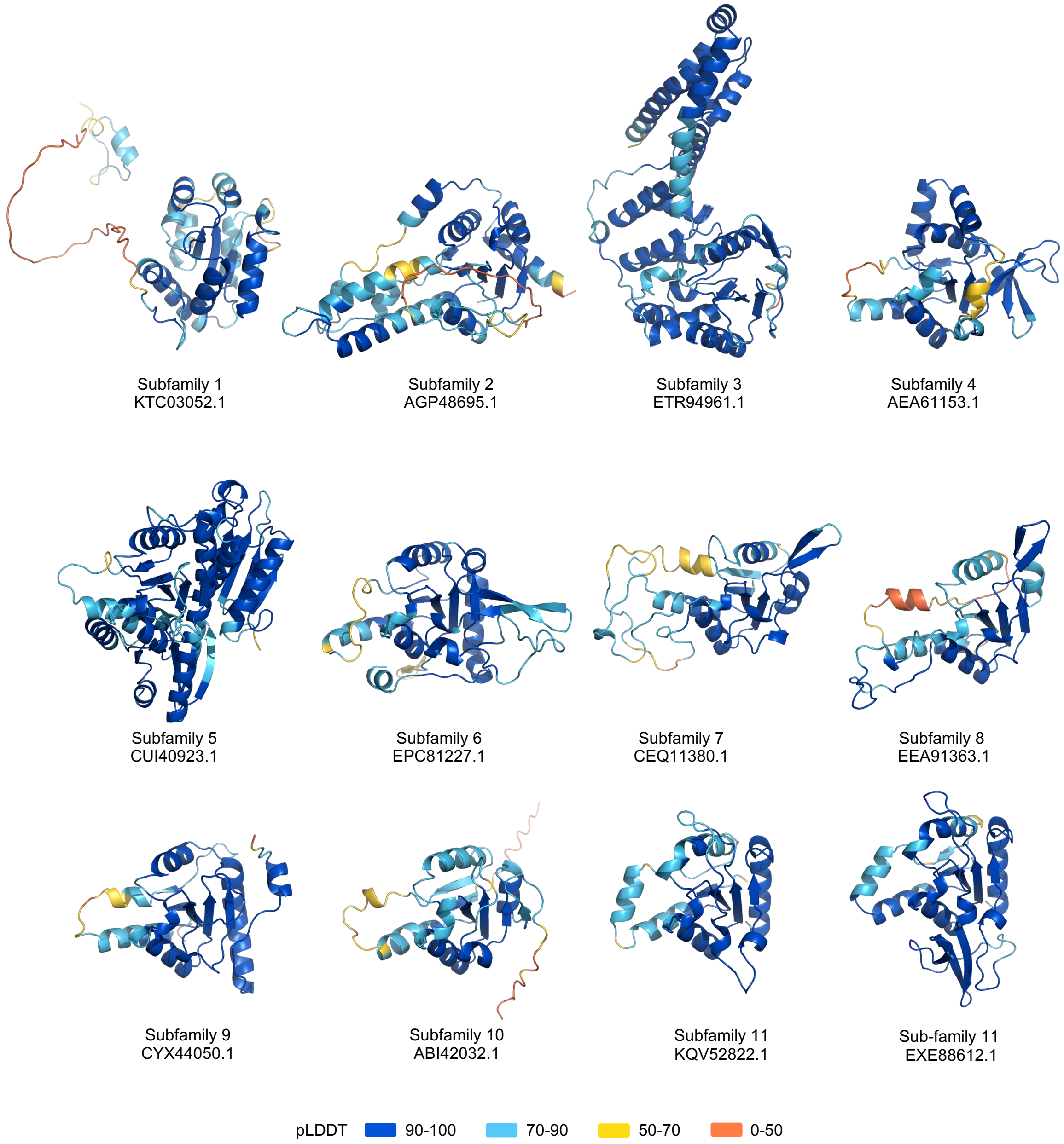

Fig. S11. Representative AlphaFold2-predicted structures of ThsB subfamilies, colored by the pLDDT score.

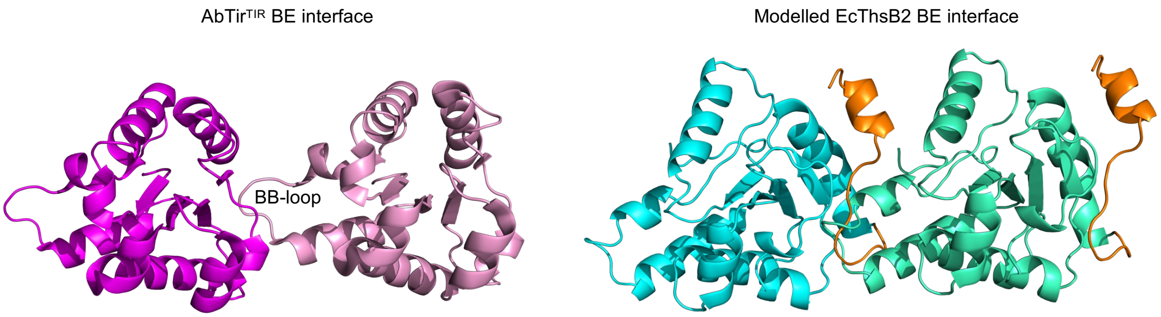

Fig. S12. Modelling of the EcThsB2 BE interface reveals steric clashes. Left panel: Ribbon representation of the BE interface (active site) in the AbTir^{TIR} filament. Right panel: Model of the EcThsB2 BE interface highlighting likely clash between the BB loop and the C-terminal tail (highlighted in orange). The model was prepared by superimposing EcThsB2 molecules onto the cryoEM structure of the AbTir^{TIR} filament (PDB: 7UXU (39)).

Table S1. Crystallographic data.

	$EcThsA^{Macro}$ (native, SIRAS)	$EcThsA^{Macro}$ (iodide derivative, SIRAS)	$EcThsA^{Macro}$ (ligand-free)	$EcThsA^{Macro}$ (ADPR complex)	$PcThsA^{Macro}$ (IAD complex)	EcThsB2 (bromide derivative)
PDB ID			8V6Q	8V6R	8V6S	8V6T
Space group	$P\ 2\ 2_1\ 2_1$	$P\ 2\ 2_1\ 2_1$	$P\ 2\ 2_1\ 2_1$	$P\ 2\ 2_1\ 2_1$	$P\ 2_1$	$P\ 4_1\ 2_1\ 2$
Cell dimensions						
a, b, c (Å)	39.24, 92.96, 134.84	39.56, 93.21, 135.28	40.04, 93.54, 140.54	39.52, 92.60, 139.98	41.86, 87.71, 65.26	72.93, 72.93, 217.54
α, β, γ (°)	90, 90, 90	90, 90, 90	90, 90, 90	90, 90, 90	90, 97, 90	90, 90, 90
Resolution (Å)	44.95 – 2.66 (2.79 – 2.66)	46.60 – 2.82 (2.97 – 2.82)	46.85 – 2.62 (2.74 – 2.62)	46.66 – 2.14 (2.20 – 2.14)	43.86 – 1.60 (1.63 – 1.60)	46.60 – 1.98 (2.03 – 1.98)
Total reflections	87,986 (10,901)	382,136 (49,843)	109,828 (12,869)	132,438 (10,448)	399,045 (20,301)	2,190,079 (144,894)
Unique reflections	14,881 (1,927)	16,538 (1,733)	16,538 (1,912)	29,133 (2,281)	60,808 (2,968)	41,733 (2,767)
Completeness (%)	99.9 (99.7)	99.6 (97.1)	99.6 (96.7)	99.8 (95.9)	98.7 (97.9)	99.7 (95.4)
Multiplicity	5.9 (5.7)	30.2 (28.8)	6.6 (6.7)	4.5 (4.6)	6.6 (6.8)	52.5 (52.4)
Wilson plot B factor ($Å^2$)			56.79	35.45	19.1	22.78
Rmeas (%)	10.2 (34.1)	17.5 (68.6)	15.5 (162.5)	11.1 (109.1)	8.9 (134.4)	13.3 (128.3)
Rmerge (%)	9.3 (30.9)	17.2 (69.6)	14.3 (150.1)	9.9 (96.9)	8.1 (124.2)	13.2 (127.1)
Rpim (%)	4.2 (14.1)	3.1 (12.8)	5.9 (61.5)	5.0 (49.0)	3.5 (51.1)	1.8 (17.3)
$\langle I/\sigma(I)\rangle$	11.0 (4.5)	18.5 (5.2)	1.4 (3.1)	9.0 (1.4)	11.3 (1.5)	35.5 (4.6)
$CC_{1/2}$	0.994 (0.960)	0.999 (0.977)	0.997 (0.525)	0.998 (0.667)	0.998 (0.770)	1.000 (0.912)
Resolution (Å)			44.38-2.62	41.67-2.14	41.53 – 1.60	46.60– 1.98
Number of reflections used			16,477	29,060	60,651	40,279
Rwork (%)			24.12	20.50	19.24	18.36
Rfree (%)			26.85	23.41	21.88	22.62
Final model						
Number of protein residues			421	423	422	485
Number of water molecules			2	41	318	246
Number of ligand atoms				72	80	7
Average B-factor ($Å^2$)						
Protein			63	59	28	31
Solvent			43	49	34	34
Ligands				ADPR: 43	IAD: 20	Br: 36

R.m.s. deviations						
Bond lengths (Å)			0.003	0.003	0.010	0.011
Bond angles (°)			0.529	0.586	1.089	0.987
Ramachandran plot (%)						
Favoured			94.24	95.23	96.65	97.53
Allowed			5.76	4.77	2.87	2.47
Outliers			0	0	0.48	0

The statistics were calculated using AIMLESS (*75*) and MolProbity(*83*). Statistics for the highest-resolution shell are shown in parentheses. $R_{merge} = \sum_{hkl}\sum_{j}|I_{hkl,j} - < I_{hkl} > |/(\sum_{hkl}\sum_{j}I_{hkl,j})$. R_{work} / $R_{free} = \sum_{hkl}\left|F_{hkl}^{obs} - F_{hkl}^{calc}\right|/(\sum_{hkl}F_{hkl}^{obs})$; R_{free} was calculated using randomly chosen 4.8-5.1 % fraction of data that was excluded from refinement.

Table S2 (Microsoft Excel format). ThsB proteins modelled by AlphaFold2.

REFERENCES AND NOTES

1. A. Bernheim, R. Sorek, The pan-immune system of bacteria: Antiviral defence as a community resource. *Nat. Rev. Microbiol.* **18**, 113–119 (2020).

2. H. G. Hampton, B. N. J. Watson, P. C. Fineran, The arms race between bacteria and their phage foes. *Nature* **577**, 327–336 (2020).

3. S. Doron, S. Melamed, G. Ofir, A. Leavitt, A. Lopatina, M. Keren, G. Amitai, R. Sorek, Systematic discovery of antiphage defense systems in the microbial pangenome. *Science* **359**, (2018).

4. L. Gao, H. Altae-Tran, F. Bohning, K. S. Makarova, M. Segel, J. L. Schmid-Burgk, J. Koob, Y. I. Wolf, E. V. Koonin, F. Zhang, Diverse enzymatic activities mediate antiviral immunity in prokaryotes. *Science* **369**, 1077–1084 (2020).

5. M. LeRoux, M. T. Laub, Toxin-antitoxin systems as phage defense elements. *Annu. Rev. Microbiol.* **76**, 21–43 (2022).

6. A. Millman, S. Melamed, A. Leavitt, S. Doron, A. Bernheim, J. Hor, J. Garb, N. Bechon, A. Brandis, A. Lopatina, G. Ofir, D. Hochhauser, A. Stokar-Avihail, N. Tal, S. Sharir, M. Voichek, Z. Erez, J. L. M. Ferrer, D. Dar, A. Kacen, G. Amitai, R. Sorek, An expanded arsenal of immune systems that protect bacteria from phages. *Cell Host Microbe* **30**, 1556–1569.e5 (2022).

7. F. Rousset, F. Depardieu, S. Miele, J. Dowding, A. L. Laval, E. Lieberman, D. Garry, E. P. C. Rocha, A. Bernheim, D. Bikard, Phages and their satellites encode hotspots of antiviral systems. *Cell Host Microbe* **30**, 740–753.e5 (2022).

8. C. N. Vassallo, C. R. Doering, M. L. Littlehale, G. I. C. Teodoro, M. T. Laub, A functional selection reveals previously undetected anti-phage defence systems in the E. coli pangenome. *Nat. Microbiol.* **7**, 1568–1579 (2022).

9. G. Hogrel, A. Guild, S. Graham, H. Rickman, S. Gruschow, Q. Bertrand, L. Spagnolo, M. F. White, Cyclic nucleotide-induced helical structure activates a TIR immune effector. *Nature* **608**, 808–812 (2022).

10. B. R. Morehouse, A. A. Govande, A. Millman, A. F. A. Keszei, B. Lowey, G. Ofir, S. Shao, R. Sorek, P. J. Kranzusch, STING cyclic dinucleotide sensing originated in bacteria. *Nature* **586**, 429–433 (2020).

11. G. Ofir, E. Herbst, M. Baroz, D. Cohen, A. Millman, S. Doron, N. Tal, D. B. A. Malheiro, S. Malitsky, G. Amitai, R. Sorek, Antiviral activity of bacterial TIR domains via immune signalling molecules. *Nature* **600**, 116–120 (2021).

12. N. Tal, B. R. Morehouse, A. Millman, A. Stokar-Avihail, C. Avraham, T. Fedorenko, E. Yirmiya, E. Herbst, A. Brandis, T. Mehlman, Y. Oppenheimer-Shaanan, A. F. A. Keszei, S. Shao, G. Amitai, P. J. Kranzusch, R. Sorek, Cyclic CMP and cyclic UMP mediate bacterial immunity against phages. *Cell* **184**, 5728–5739.e16 (2021).

13. A. T. Whiteley, J. B. Eaglesham, C. C. de Oliveira Mann, B. R. Morehouse, B. Lowey, E. A. Nieminen, O. Danilchanka, D. S. King, A. S. Y. Lee, J. J. Mekalanos, P. J. Kranzusch, Bacterial cGAS-like enzymes synthesize diverse nucleotide signals. *Nature* **567**, 194–199 (2019).

14. J. Garb, A. Lopatina, A. Bernheim, M. Zaremba, V. Siksnys, S. Melamed, A. Leavitt, A. Millman, G. Amitai, R. Sorek, Multiple phage resistance systems inhibit infection via SIR2-dependent NAD(+) depletion. *Nat. Microbiol.* **7**, 1849–1856 (2022).

15. B. Koopal, A. Potocnik, S. K. Mutte, C. Aparicio-Maldonado, S. Lindhoud, J. J. M. Vervoort, S. J. J. Brouns, D. C. Swarts, Short prokaryotic Argonaute systems trigger cell death upon detection of invading DNA. *Cell* **185**, 1471–1486.e19 (2022).

16. B. Y. Hsueh, G. B. Severin, C. A. Elg, E. J. Waldron, A. Kant, A. J. Wessel, J. A. Dover, C. R. Rhoades, B. J. Ridenhour, K. N. Parent, M. B. Neiditch, J. Ravi, E. M. Top, C. M. Waters, Phage defence by deaminase-mediated depletion of deoxynucleotides in bacteria. *Nat. Microbiol.* **7**, 1210–1220 (2022).

17. N. Tal, A. Millman, A. Stokar-Avihail, T. Fedorenko, A. Leavitt, S. Melamed, E. Yirmiya, C. Avraham, A. Brandis, T. Mehlman, G. Amitai, R. Sorek, Bacteria deplete deoxynucleotides to defend against bacteriophage infection. *Nat. Microbiol.* **7**, 1200–1209 (2022).

18. B. Duncan-Lowey, N. Tal, A. G. Johnson, S. Rawson, M. L. Mayer, S. Doron, A. Millman, S. Melamed, T. Fedorenko, A. Kacen, A. Brandis, T. Mehlman, G. Amitai, R. Sorek, P. J. Kranzusch, Cryo-EM structure of the RADAR supramolecular anti-phage defense complex. *Cell* **186**, 987–998.e15 (2023).

19. M. LeRoux, S. Srikant, G. I. C. Teodoro, T. Zhang, M. L. Littlehale, S. Doron, M. Badiee, A. K. L. Leung, R. Sorek, M. T. Laub, The DarTG toxin-antitoxin system provides phage defence by ADP-ribosylating viral DNA. *Nat. Microbiol.* **7**, 1028–1040 (2022).

20. B. Duncan-Lowey, N. K. McNamara-Bordewick, N. Tal, R. Sorek, P. J. Kranzusch, Effector-mediated membrane disruption controls cell death in CBASS antiphage defense. *Mol. Cell* **81**, 5039–5051.e5 (2021).

21. A. G. Johnson, T. Wein, M. L. Mayer, B. Duncan-Lowey, E. Yirmiya, Y. Oppenheimer-Shaanan, G. Amitai, R. Sorek, P. J. Kranzusch, Bacterial gasdermins reveal an ancient mechanism of cell death. *Science* **375**, 221–225 (2022).

22. A. Bernheim, A. Millman, G. Ofir, G. Meitav, C. Avraham, H. Shomar, M. M. Rosenberg, N. Tal, S. Melamed, G. Amitai, R. Sorek, Prokaryotic viperins produce diverse antiviral molecules. *Nature* **589**, 120–124 (2021).

23. L. Kever, A. Hardy, T. Luthe, M. Hunnefeld, C. Gatgens, L. Milke, J. Wiechert, J. Wittmann, C. Moraru, J. Marienhagen, J. Frunzke, Aminoglycoside antibiotics inhibit phage infection by blocking an early step of the infection cycle. *MBio* **13**, e0078322 (2022).

24. S. Kronheim, M. Daniel-Ivad, Z. Duan, S. Hwang, A. I. Wong, I. Mantel, J. R. Nodwell, K. L. Maxwell, A chemical defence against phage infection. *Nature* **564**, 283–286 (2018).

25. J. Bobonis, K. Mitosch, A. Mateus, N. Karcher, G. Kritikos, J. Selkrig, M. Zietek, V. Monzon, B. Pfalz, S. Garcia-Santamarina, M. Galardini, A. Sueki, C. Kobayashi, F. Stein, A. Bateman, G. Zeller, M. M. Savitski, J. R. Elfenbein, H. L. Andrews-Polymenis, A. Typas, Bacterial retrons encode phage-defending tripartite toxin-antitoxin systems. *Nature* **609**, 144–150 (2022).

26. A. Millman, A. Bernheim, A. Stokar-Avihail, T. Fedorenko, M. Voichek, A. Leavitt, Y. Oppenheimer-Shaanan, R. Sorek, Bacterial retrons function in anti-phage defense. *Cell* **183**, 1551–1561.e12 (2020).

27. K. Essuman, J. Milbrandt, J. L. Dangl, M. T. Nishimura, Shared TIR enzymatic functions regulate cell death and immunity across the tree of life. *Science* **377**, eabo0001 (2022).

28. S. Li, M. K. Manik, Y. Shi, B. Kobe, T. Ve, Toll/interleukin-1 receptor domains in bacterial and plant immunity. *Curr. Opin. Microbiol.* **74**, 102316 (2023).

29. S. Nimma, W. Gu, N. Maruta, Y. Li, M. Pan, F. K. Saikot, B. Y. J. Lim, H. Y. McGuinness, Z. F. Zaoti, S. Li, S. Desa, M. K. Manik, J. D. Nanson, B. Kobe, Structural evolution of TIR-domain signalosomes. *Front. Immunol.* **12**, 784484 (2021).

30. K. A. Fitzgerald, J. C. Kagan, Toll-like Receptors and the Control of Immunity. *Cell* **180**, 1044–1066 (2020).

31. L. A. O'Neill, D. Golenbock, A. G. Bowie, The history of Toll-like receptors - redefining innate immunity. *Nat. Rev. Immunol.* **13**, 453–460 (2013).

32. M. T. B. Clabbers, S. Holmes, T. W. Muusse, P. R. Vajjhala, S. J. Thygesen, A. K. Malde, D. J. B. Hunter, T. I. Croll, L. Flueckiger, J. D. Nanson, M. H. Rahaman, A. Aquila, M. S. Hunter, M. Liang, C. H. Yoon, J. Zhao, N. A. Zatsepin, B. Abbey, E. Sierecki, Y. Gambin, K. J. Stacey, C. Darmanin, B. Kobe, H. Xu, T. Ve, MyD88 TIR domain higher-order assembly interactions revealed by microcrystal electron diffraction and serial femtosecond crystallography. *Nat. Commun.* **12**, 2578 (2021).

33. T. Ve, P. R. Vajjhala, A. Hedger, T. Croll, F. DiMaio, S. Horsefield, X. Yu, P. Lavrencic, Z. Hassan, G. P. Morgan, A. Mansell, M. Mobli, A. O'Carroll, B. Chauvin, Y. Gambin, E. Sierecki, M. J. Landsberg, K. J. Stacey, E. H. Egelman, B. Kobe, Structural basis of TIR-domain-assembly formation in MAL- and MyD88-dependent TLR4 signaling. *Nat. Struct. Mol. Biol.* **24**, 743–751 (2017).

34. K. Essuman, D. W. Summers, Y. Sasaki, X. Mao, A. DiAntonio, J. Milbrandt, The SARM1 Toll/Interleukin-1 receptor domain possesses intrinsic NAD(+) cleavage activity that promotes pathological axonal degeneration. *Neuron* **93**, 1334–1343.e5 (2017).

35. K. Essuman, D. W. Summers, Y. Sasaki, X. Mao, A. K. Y. Yim, A. DiAntonio, J. Milbrandt, TIR domain proteins are an ancient family of NAD(+)-consuming enzymes. *Curr. Biol.* **28**, 421–430.e4 (2018).

36. S. Horsefield, H. Burdett, X. Zhang, M. K. Manik, Y. Shi, J. Chen, T. Qi, J. Gilley, J. S. Lai, M. X. Rank, L. W. Casey, W. Gu, D. J. Ericsson, G. Foley, R. O. Hughes, T. Bosanac, M. von Itzstein, J. P. Rathjen, J. D. Nanson, M. Boden, I. B. Dry, S. J. Williams, B. J. Staskawicz, M. P. Coleman, T. Ve, P. N. Dodds, B. Kobe, NAD(+) cleavage activity by animal and plant TIR domains in cell death pathways. *Science* **365**, 793–799 (2019).

37. L. Wan, K. Essuman, R. G. Anderson, Y. Sasaki, F. Monteiro, E. H. Chung, E. Osborne Nishimura, A. DiAntonio, J. Milbrandt, J. L. Dangl, M. T. Nishimura, TIR domains of plant immune receptors are NAD(+)-cleaving enzymes that promote cell death. *Science* **365**, 799–803 (2019).

38. A. Leavitt, E. Yirmiya, G. Amitai, A. Lu, J. Garb, E. Herbst, B. R. Morehouse, S. J. Hobbs, S. P. Antine, Z. J. Sun, P. J. Kranzusch, R. Sorek, Viruses inhibit TIR gcADPR signalling to overcome bacterial defence. *Nature* **611**, 326–331 (2022).

39. M. K. Manik, Y. Shi, S. Li, M. A. Zaydman, N. Damaraju, S. Eastman, T. G. Smith, W. Gu, V. Masic, T. Mosaiab, J. S. Weagley, S. J. Hancock, E. Vasquez, L. Hartley-Tassell, N. Kargios, N. Maruta, B. Y. J. Lim, H. Burdett, M. J. Landsberg, M. A. Schembri, I. Prokes, L. Song, M. Grant, A. DiAntonio, J. D. Nanson, M. Guo, J. Milbrandt, T. Ve, B. Kobe, Cyclic ADP ribose isomers: Production, chemical structures, and immune signaling. *Science* **377**, eadc8969 (2022).

40. S. Huang, A. Jia, W. Song, G. Hessler, Y. Meng, Y. Sun, L. Xu, H. Laessle, J. Jirschitzka, S. Ma, Y. Xiao, D. Yu, J. Hou, R. Liu, H. Sun, X. Liu, Z. Han, J. Chang, J. E. Parker, J. Chai, Identification and receptor mechanism of TIR-catalyzed small molecules in plant immunity. *Science* **377**, eabq3297 (2022).

41. A. Jia, S. Huang, W. Song, J. Wang, Y. Meng, Y. Sun, L. Xu, H. Laessle, J. Jirschitzka, J. Hou, T. Zhang, W. Yu, G. Hessler, E. Li, S. Ma, D. Yu, J. Gebauer, U. Baumann, X. Liu, Z. Han, J. Chang, J. E. Parker, J. Chai, TIR-catalyzed ADP-ribosylation reactions produce signaling molecules for plant immunity. *Science* **377**, eabq8180 (2022).

42. S. Imai, C. M. Armstrong, M. Kaeberlein, L. Guarente, Transcriptional silencing and longevity protein Sir2 is an NAD-dependent histone deacetylase. *Nature* **403**, 795–800 (2000).

43. J. C. Tanny, G. J. Dowd, J. Huang, H. Hilz, D. Moazed, An enzymatic activity in the yeast Sir2 protein that is essential for gene silencing. *Cell* **99**, 735–745 (1999).

44. D. Ahel, Z. Horejsi, N. Wiechens, S. E. Polo, E. Garcia-Wilson, I. Ahel, H. Flynn, M. Skehel, S. C. West, S. P. Jackson, T. Owen-Hughes, S. J. Boulton, Poly(ADP-ribose)-dependent regulation of DNA repair by the chromatin remodeling enzyme ALC1. *Science* **325**, 1240–1243 (2009).

45. G. I. Karras, G. Kustatscher, H. R. Buhecha, M. D. Allen, C. Pugieux, F. Sait, M. Bycroft, A. G. Ladurner, The macro domain is an ADP-ribose binding module. *EMBO J.* **24**, 1911–1920 (2005).

46. W. L. Kraus, New functions for an ancient domain. *Nat. Struct. Mol. Biol.* **16**, 904–907 (2009).

47. D. Slade, M. S. Dunstan, E. Barkauskaite, R. Weston, P. Lafite, N. Dixon, M. Ahel, D. Leys, I. Ahel, The structure and catalytic mechanism of a poly(ADP-ribose) glycohydrolase. *Nature* **477**, 616–620 (2011).

48. G. Jankevicius, M. Hassler, B. Golia, V. Rybin, M. Zacharias, G. Timinszky, A. G. Ladurner, A family of macrodomain proteins reverses cellular mono-ADP-ribosylation. *Nat. Struct. Mol. Biol.* **20**, 508–514 (2013).

49. C. Li, Y. Debing, G. Jankevicius, J. Neyts, I. Ahel, B. Coutard, B. Canard, Viral macro domains reverse protein ADP-ribosylation. *J. Virol.* **90**, 8478–8486 (2016).

50. F. Rosenthal, K. L. Feijs, E. Frugier, M. Bonalli, A. H. Forst, R. Imhof, H. C. Winkler, D. Fischer, A. Caflisch, P. O. Hassa, B. Luscher, M. O. Hottiger, Macrodomain-containing proteins are new mono-ADP-ribosylhydrolases. *Nat. Struct. Mol. Biol.* **20**, 502–507 (2013).

51. D. Ka, H. Oh, E. Park, J. H. Kim, E. Bae, Structural and functional evidence of bacterial antiphage protection by Thoeris defense system via NAD^+ degradation. *Nat. Commun.* **11**, 2816 (2020).

52. L. A. Stevens, J. Kato, A. Kasamatsu, H. Oda, D. Y. Lee, J. Moss, The ARH and macrodomain families of α-ADP-ribose-acceptor hydrolases catalyze α-NAD^+ Hydrolysis. *ACS Chem. Biol.* **14**, 2576–2584 (2019).

53. S. Wazir, M. M. Maksimainen, H. I. Alanen, A. Galera-Prat, L. Lehtio, Activity-based screening assay for mono-ADP-ribosylhydrolases. *SLAS Discov.* **26**, 67–76 (2021).

54. L. Holm, P. Rosenstrom, Dali server: Conservation mapping in 3D. *Nucleic Acids Res.* **38**, W545–549 (2010).

55. R. Zapata-Perez, F. Gil-Ortiz, A. B. Martinez-Monino, A. G. Garcia-Saura, J. Juanhuix, A. Sanchez-Ferrer, Structural and functional analysis of Oceanobacillus iheyensis macrodomain reveals a network of waters involved in substrate binding and catalysis. *Open Biol.* **7**, 160327 (2017).

56. A. H. Forst, T. Karlberg, N. Herzog, A. G. Thorsell, A. Gross, K. L. Feijs, P. Verheugd, P. Kursula, B. Nijmeijer, E. Kremmer, H. Kleine, A. G. Ladurner, H. Schuler, B. Luscher, Recognition of mono-ADP-ribosylated ARTD10 substrates by ARTD8 macrodomains. *Structure* **21**, 462–475 (2013).

57. G. Timinszky, S. Till, P. O. Hassa, M. Hothorn, G. Kustatscher, B. Nijmeijer, J. Colombelli, M. Altmeyer, E. H. Stelzer, K. Scheffzek, M. O. Hottiger, A. G. Ladurner, A macrodomain-containing histone rearranges chromatin upon sensing PARP1 activation. *Nat. Struct. Mol. Biol.* **16**, 923–929 (2009).

58. Y. Shi, P. S. Kerry, J. D. Nanson, T. Bosanac, Y. Sasaki, R. Krauss, F. K. Saikot, S. E. Adams, T. Mosaiab, V. Masic, X. Mao, F. Rose, E. Vasquez, M. Furrer, K. Cunnea, A. Brearley, W. Gu, Z. Luo, L. Brillault, M. J. Landsberg, A. DiAntonio, B. Kobe, J. Milbrandt, R. O. Hughes, T. Ve, Structural basis of SARM1 activation, substrate recognition, and inhibition by small molecules. *Mol. Cell* **82**, 1643–1659.e10 (2022).

59. S. R. Armstrong, W. J. Cook, S. A. Short, S. E. Ealick, Crystal structures of nucleoside 2-deoxyribosyltransferase in native and ligand-bound forms reveal architecture of the active site. *Structure* **4**, 97–107 (1996).

60. Y. K. Ghiorghi, K. I. Zeller, C. V. Dang, P. A. Kaminski, The c-Myc target gene Rcl (C6orf108) encodes a novel enzyme, deoxynucleoside 5′-monophosphate N-glycosidase. *J. Biol. Chem.* **282**, 8150–8156 (2007).

61. M. D. Sikowitz, L. E. Cooper, T. P. Begley, P. A. Kaminski, S. E. Ealick, Reversal of the substrate specificity of CMP N-glycosidase to dCMP. *Biochemistry* **52**, 4037–4047 (2013).

62. A. M. Bayless, S. Chen, S. C. Ogden, X. Xu, J. D. Sidda, M. K. Manik, S. Li, B. Kobe, T. Ve, L. Song, M. Grant, L. Wan, M. T. Nishimura, Plant and prokaryotic TIR domains generate distinct cyclic ADPR NADase products. *Sci. Adv.* **9**, eade8487 (2023).

63. N. Maruta, M. Sorbello, B. Y. J. Lim, H. Y. McGuinness, Y. Shi, T. Ve, B. Kobe, TIR domain-associated nucleotides with functions in plant immunity and beyond. *Curr. Opin. Plant Biol.* **73**, 102364 (2023).

64. J. G. Rack, D. Perina, I. Ahel, Macrodomains: Structure, function, evolution, and catalytic activities. *Annu. Rev. Biochem.* **85**, 431–454 (2016).

65. K. Doddapaneni, B. Mahler, R. Pavlovicz, A. Haushalter, C. Yuan, Z. Wu, Solution structure of RCL, a novel 2′-deoxyribonucleoside 5′-monophosphate N-glycosidase. *J. Mol. Biol.* **394**, 423–434 (2009).

66. A. M. Pedley, S. J. Benkovic, A new view into the regulation of purine metabolism: The purinosome. *Trends Biochem. Sci.* **42**, 141–154 (2017).

67. A. Koh, A. Molinaro, M. Stahlman, M. T. Khan, C. Schmidt, L. Manneras-Holm, H. Wu, A. Carreras, H. Jeong, L. E. Olofsson, P. O. Bergh, V. Gerdes, A. Hartstra, M. de Brauw, R. Perkins, M. Nieuwdorp, G. Bergstrom, F. Backhed, Microbially produced imidazole propionate impairs insulin signaling through mTORC1. *Cell* **175**, 947–961.e17 (2018).

68. M. Kohlmeier, “Amino acids and nitrogen compounds” in Nutrient Metabolism: Structures, Functions, and Genes (Elsevier, ed. 2, 2015), pp. 431–439.

69. D. Sabonis, C. Avraham, A. Lu, E. Herbst, A. Silanskas, A. Leavitt, E. Yirmiya, M. Zaremba, G. Amitai, P. J. Kranzusch, R. Sorek, G. Tamulaitiene, TIR domains produce histidine-ADPR conjugates as immune signaling molecules in bacteria. bioRxiv 573942 [Preprint] (2024). https://doi.org/10.1101/2024.01.03.573942.

70. M. D. Figley, W. Gu, J. D. Nanson, Y. Shi, Y. Sasaki, K. Cunnea, A. K. Malde, X. Jia, Z. Luo, F. K. Saikot, T. Mosaiab, V. Masic, S. Holt, L. Hartley-Tassell, H. Y. McGuinness, M. K. Manik, T. Bosanac, M. J. Landsberg, P. S. Kerry, M. Mobli, R. O. Hughes, J. Milbrandt, B. Kobe, A. DiAntonio, T. Ve, SARM1 is a metabolic sensor activated by an increased NMN/NAD^+ ratio to trigger axon degeneration. *Neuron* **109**, 1118–1136.e11 (2021).

71. B. R. Morehouse, M. C. J. Yip, A. F. A. Keszei, N. K. McNamara-Bordewick, S. Shao, P. J. Kranzusch, Cryo-EM structure of an active bacterial TIR-STING filament complex. *Nature* **608**, 803–807 (2022).

72. L. Stols, M. Gu, L. Dieckman, R. Raffen, F. R. Collart, M. I. Donnelly, A new vector for high-throughput, ligation-independent cloning encoding a tobacco etch virus protease cleavage site. *Protein Expr. Purif.* **25**, 8–15 (2002).

73. F. W. Studier, Protein production by auto-induction in high-density shaking cultures. *Protein Expr. Purif.* **41**, 207–234 (2005).

74. W. Kabsch, XDS *Acta Crystallogr. D Biol. Crystallogr.* **66** (Pt. 2), 125–132 (2010).

75. P. Evans, Scaling and assessment of data quality. *Acta Crystallogr. D Biol. Crystallogr.* **62** (Pt. 1), 72–82 (2006).

76. N. S. Pannu, W. J. Waterreus, P. Skubak, I. Sikharulidze, J. P. Abrahams, R. A. de Graaff, Recent advances in the CRANK software suite for experimental phasing. *Acta Crystallogr. D Biol. Crystallogr.* **67** (Pt. 4), 331–337 (2011).

77. G. M. Sheldrick, A short history ofSHELX. *Acta Crystallogr. A* **64**, 112–122 (2008).

78. K. Cowtan, The Buccaneer software for automated model building. 1. Tracing protein chains. *Acta Crystallogr. D Biol. Crystallogr.* **62** (Pt. 9), 1002–1011 (2006).

79. G. N. Murshudov, P. Skubak, A. A. Lebedev, N. S. Pannu, R. A. Steiner, R. A. Nicholls, M. D. Winn, F. Long, A. A. Vagin, REFMAC5 for the refinement of macromolecular crystal structures. *Acta Crystallogr. D Biol. Crystallogr.* **67** (Pt. 4), 355–367 (2011).

80. A. J. McCoy, Solving structures of protein complexes by molecular replacement with Phaser. *Acta Crystallogr. D Biol. Crystallogr.* **63** (Pt. 1), 32–41 (2007).

81. P. D. Adams, D. Baker, A. T. Brunger, R. Das, F. DiMaio, R. J. Read, D. C. Richardson, J. S. Richardson, T. C. Terwilliger, Advances, interactions, and future developments in the CNS, Phenix, and Rosetta structural biology software systems. *Annu. Rev. Biophys.* **42**, 265–287 (2013).

82. P. Emsley, B. Lohkamp, W. G. Scott, K. Cowtan, Features and development of Coot. *Acta Crystallogr. D Biol. Crystallogr.* **66** (Pt. 4), 486–501 (2010).

83. V. B. Chen, W. B. Arendall III, J. J. Headd, D. A. Keedy, R. M. Immormino, G. J. Kapral, L. W. Murray, J. S. Richardson, D. C. Richardson, MolProbity: All-atom structure validation for macromolecular crystallography. *Acta Crystallogr. D Biol. Crystallogr.* **66** (Pt. 1), 12–21 (2010).

84. J. Jumper, R. Evans, A. Pritzel, T. Green, M. Figurnov, O. Ronneberger, K. Tunyasuvunakool, R. Bates, A. Zidek, A. Potapenko, A. Bridgland, C. Meyer, S. A. A. Kohl, A. J. Ballard, A. Cowie, B. Romera-Paredes, S. Nikolov, R. Jain, J. Adler, T. Back, S. Petersen, D. Reiman, E. Clancy, M. Zielinski, M. Steinegger, M. Pacholska, T. Berghammer, S. Bodenstein, D. Silver, O. Vinyals, A. W. Senior, K. Kavukcuoglu, P. Kohli, D. Hassabis, Highly accurate protein structure prediction with AlphaFold. *Nature* **596**, 583–589 (2021).

85. G. M. Sheldrick, Experimental phasing with SHELXC/D/E: Combining chain tracing with density modification. *Acta Crystallogr. D Biol. Crystallogr.* **66** (Pt. 4), 479–485 (2010).

86. R. Evans, M. O'Neill, A. Pritzel, N. Antropova, A. Senior, T. Green, A. Žídek, R. Bates, S. Blackwell, J. Yim, O. Ronneberger, S. Bodenstein, M. Zielinski, A. Bridgland, A. Potapenko, A. Cowie, K. Tunyasuvunakool, R. Jain, E. Clancy, P. Kohli, J. Jumper, D. Hassabis, Protein complex prediction

with AlphaFold-Multimer. bioRxiv 463034 [Preprint] (2022). https://doi.org/10.1101/2021.10.04.463034.

87. M. Mirdita, K. Schutze, Y. Moriwaki, L. Heo, S. Ovchinnikov, M. Steinegger, ColabFold: Making protein folding accessible to all. *Nat. Methods* **19**, 679–682 (2022).

88. W. Li, A. Godzik, Cd-hit: A fast program for clustering and comparing large sets of protein or nucleotide sequences. *Bioinformatics* **22**, 1658–1659 (2006).

89. D. Moi, C. Bernard, M. Steinegger, Y. Nevers, M. Langleib, C. Dessimoz, Structural phylogenetics unravels the evolutionary diversification of communication systems in gram-positive bacteria and their viruses. bioRxiv 558401 [Preprint] (2023). https://doi.org/10.1101/2023.09.19.558401.

90. I. Letunic, P. Bork, Interactive Tree Of Life (iTOL) v5: An online tool for phylogenetic tree display and annotation. *Nucleic Acids Res.* **49**, W293–W296 (2021).

91. M. Piotto, V. Saudek, V. Sklenar, Gradient-tailored excitation for single-quantum NMR spectroscopy of aqueous solutions. *J. Biomol. NMR* **2**, 661–665 (1992).

92. V. Sklenar, M. Piotto, R. Leppik, V. Saudek, Gradient-tailored water suppression for 1H-15N HSQC experiments optimized to retain full sensitivity. *J. Magn. Reson.* **102**, 241–245 (1993).

93. M. Mayer, B. Meyer, Characterization of ligand binding by saturation transfer difference NMR spectroscopy. *Angew. Chem. Int. Ed. Engl.* **38**, 1784–1788 (1999).

94. H. Ashkenazy, S. Abadi, E. Martz, O. Chay, I. Mayrose, T. Pupko, N. Ben-Tal, ConSurf 2016: An improved methodology to estimate and visualize evolutionary conservation in macromolecules. *Nucleic Acids Res.* **44**, W344–350 (2016).

95. S. Ma, D. Lapin, L. Liu, Y. Sun, W. Song, X. Zhang, E. Logemann, D. Yu, J. Wang, J. Jirschitzka, Z. Han, P. Schulze-Lefert, J. E. Parker, J. Chai, Direct pathogen-induced assembly of an NLR immune receptor complex to form a holoenzyme. *Science* **370**, eabe3069 (2020).

96. A. Padilla, C. Amiable, S. Pochet, P. A. Kaminski, G. Labesse, Structure of the oncoprotein Rcl bound to three nucleotide analogues. *Acta Crystallogr. D Biol. Crystallogr.* **69** (Pt. 2), 247–255 (2013).

97. X. Robert, P. Gouet, Deciphering key features in protein structures with the new ENDscript server. *Nucleic Acids Res.* **42**, W320–W324 (2014).

98. Z. Shen, X. Y. Yang, S. Xia, W. Huang, D. J. Taylor, K. Nakanishi, T. M. Fu, Oligomerization-mediated activation of a short prokaryotic Argonaute. *Nature* **621**, 154–161 (2023).

99. R. Martin, T. Qi, H. Zhang, F. Liu, M. King, C. Toth, E. Nogales, B. J. Staskawicz, Structure of the activated ROQ1 resistosome directly recognizing the pathogen effector XopQ. *Science* **370**, eabd9993 (2020).

100. S. J. Williams, K. H. Sohn, L. Wan, M. Bernoux, P. F. Sarris, C. Segonzac, T. Ve, Y. Ma, S. B. Saucet, D. J. Ericsson, L. W. Casey, T. Lonhienne, D. J. Winzor, X. Zhang, A. Coerdt, J. E. Parker, P. N. Dodds, B. Kobe, J. D. Jones, Structural basis for assembly and function of a heterodimeric plant immune receptor. *Science* **344**, 299–303 (2014).

101. M. Bernoux, T. Ve, S. Williams, C. Warren, D. Hatters, E. Valkov, X. Zhang, J. G. Ellis, B. Kobe, P. N. Dodds, Structural and functional analysis of a plant resistance protein TIR domain reveals interfaces for self-association, signaling, and autoregulation. *Cell Host Microbe* **9**, 200–211 (2011).

102. S. Devi, A. E. Carberry, G. M. Zickuhr, A. L. Dickson, D. J. Harrison, R. G. da Silva, Human 2′-deoxynucleoside 5′-PhosphateN-Hydrolase 1: Mechanism of 2′-deoxyuridine 5′-monophosphate hydrolysis. *Biochemistry* **62**, 2658–2668 (2023).
